# Behavioral nudges prevent loan delinquencies at scale: A 13-million-person field experiment

**DOI:** 10.1073/pnas.2416708122

**Published:** 2025-01-23

**Authors:** Robert Kuan, Kristin Blagg, Benjamin L. Castleman, Rajeev Darolia, Jordan D. Matsudaira, Katherine L. Milkman, Lesley J. Turner

**Affiliations:** ^a^Department of Operations, Information and Decisions, The Wharton School, University of Pennsylvania, Philadelphia, PA 19104; ^b^Center on Education Data and Policy, The Urban Institute, Washington, DC 20024; ^c^The Frank Batten School of Leadership and Public Policy, University of Virginia, Charlottesville, VA 22904; ^d^Office of the Chief Economist, U.S. Department of Education, Washington, DC 20202; ^e^The James W. Martin School of Public Policy and Administration, University of Kentucky, Lexington, KY 40506; ^f^Department of Public Administration and Policy, The School of Public Affairs, American University, Washington, DC 20016; ^g^The Harris School of Public Policy, University of Chicago, Chicago, IL 60637

**Keywords:** nudges, student loans, framing, simplification, field experiment

## Abstract

Americans collectively hold over $1.6 trillion in student loan debt, and over the last decade millions of borrowers have defaulted on loans, with serious consequences for their financial health. In a 13-million-person field experiment with the U.S. Department of Education, we tested the effectiveness of different email interventions to inform borrowers about alternative repayment options after a missed loan payment. Our interventions tested whether sending monthly behaviorally-informed emails, providing follow-up reminders, framing benefits in percentage (vs. dollar) terms, and providing just one recommended action step at a time (vs. two) affected borrower outcomes. We find that i) behaviorally-informed emails reduce estimated 60-d delinquencies by 0.42 pp, ii) reminders boost the efficacy of such emails by 0.57 pp, iii) describing potential savings in percentage terms is more effective than describing these benefits in dollar terms, reducing estimated delinquencies by 0.14 pp, and iv) encouraging two actions (i.e., enrollment in income-driven repayment plans and auto debit programs) repeatedly across two emails is marginally more effective than encouraging one action at-a-time across two emails, reducing estimated delinquencies by 0.05 pp. Overall, if scaled to all 13-million borrowers in our experiment, we estimate that our best-performing intervention would have averted approximately 79,800 60-d delinquencies. Our findings i) highlight the benefits of describing potential savings in percentage terms, which may magnify perceived savings for recipients, ii) underscore the risks of oversimplification, and iii) demonstrate that nudges can be an effective, low-cost complement to other policies for reducing delinquencies and supporting borrowers with student loan debt.

## Significance

With rising levels of student loan debt, finding strategies to help students repay their loans has become a critical policy challenge. In a 13-million-person field experiment, we test whether sending behaviorally-informed emails can nudge borrowers at scale to improve repayment outcomes. We find redesigned emails reduce delinquencies by 0.42 percentage points. Importantly, we also find that describing potential savings in percentage (vs. dollar) terms and sending emails that recommend two action steps (vs. one action step) improves borrower outcomes. These findings suggest that low-cost nudges can be an effective and scalable tool to help reduce loan delinquencies and address loan repayment difficulties faced by many borrowers.

In 2023, roughly 46 million Americans held federal student loan debt, with the total value of this debt exceeding $1.6 trillion (1). Many borrowers struggle to repay their loans: roughly one in seven borrowers in repayment experienced loan delinquency or default between 2012 and 2019 (2). Student loan delinquency and default can have harmful consequences, including impaired credit ratings, wage garnishment, and offsets of tax refunds and Social Security benefits (3).

The United States Department of Education and federal loan servicers offer several programs designed to help borrowers more effectively manage and repay their loans. These include i) offering income-driven repayment (IDR) plans that provide insurance against unaffordable loan payments when income is low by linking payments to income and ii) facilitating auto debit payments so borrowers need not remember to manually pay their bill(s) each month and incentivizing borrowers to sign-up (with a 0.25 percentage point interest rate reduction).

Switching to an IDR plan has been shown to both improve student loan repayment outcomes and overall financial well-being (4). However, many borrowers who likely would benefit from lower payments on IDR do not sign up, probably due to a combination of signup hassle factors (5, 6), the confusing framing of the costs and benefits of IDR plans (7), and an aversion to thinking about student loan debt (8). Many borrowers also forgo reductions in their monthly payments by neglecting to sign up for auto debit payments. Like IDR enrollment, auto debit signup also requires overcoming hassle factors (e.g., making an active choice), and thinking about loan debt.

In this paper, we address the question of how more borrowers can be nudged to sign up for IDR plans and auto debit payments, and whether interventions designed to increase sign-ups for these programs can reduce student loan delinquencies. We also present two theory-driven tests to assess the effectiveness of individual components of behaviorally-informed emails. First, we test whether presenting substantial potential financial savings from IDR plans in dollars terms (rather than percentage terms) is effective. This approach is consistent with the theory that those low in numeracy benefit when information is presented in a more familiar format (9, 10) and the theory of mental accounting, which predicts that percentage discounts are perceived as less substantial in larger mental accounts (11), leading discounts in such accounts described in dollar terms to be perceived as more meaningful (12). Second, we test whether email communications are more effective if they are simplified to recommend one action at a time, consistent with the theory that simplification drives adoption (13).

In partnership with the U.S. Department of Education, we designed and evaluated a behaviorally-informed email campaign intended to reduce inattention to and/or misunderstandings of the benefits of signing up for i) IDR plans and ii) auto debit payments. We conducted a six-arm, randomized control trial to assess the short- and medium-term effects of sending different behaviorally-informed email messages to 13-million federal loan borrowers encouraging IDR and auto debit signup. Our experiment randomized i) whether borrowers received a behaviorally-informed email encouraging sign-up for these programs, ii) whether they received a reminder message, iii) whether the potential benefits of IDR plans were described in percentage or dollar terms, and iv) whether two separate emails encouraged both IDR application and auto debit signup or each email encouraged just one of these two actions.

Our work builds on past field research showing that behaviorally-informed communications can increase the rate at which borrowers sign up for IDR plans. Two unpublished field experiments conducted by the White House’s Social and Behavioral Sciences Team in 2013 and 2015 found that sending a single email to inform borrowers about the benefits of applying for IDR plans significantly increased IDR applications; however, these experiments did not examine the policy-relevant outcomes we study here: namely, student loan delinquency and repayment, nor are details about these studies publicly available (14, 15). Mueller and Yannelis (6) conducted a field experiment showing that sending prefilled IDR applications to a narrow borrower population—those who called in for assistance and consulted with a repayment plan specialist about their payment options—significantly increased IDR enrollment and reduced delinquencies. Our study builds on these findings and presents field experimental evidence at a national scale, involving millions of borrowers, to establish that behaviorally-informed communications can support better borrower outcomes. We also build on a larger body of research suggesting that behaviorally-informed reminders designed to reduce forgetfulness and simplify complex information can improve policy-relevant outcomes in other domains, improving outcomes such as on-time court appearances (16), vaccination rates (17–20), the claiming of tax credits (21), and adoption of Supplemental Nutrition Assistance Program (SNAP) benefits (22).

In addition to assessing the impact of simplified, behaviorally-informed messaging and, more generally, the benefits of reminders, we examine two theory-driven questions regarding the most effective way to encourage signups for IDR plans and auto debit payments. First, we offer a test of whether it is more motivating to explain the potential savings borrowers could gain from signing up for an IDR plan in dollar or percentage terms. Prior laboratory research on consumer perceptions suggests that describing large financial benefits in dollar terms (e.g., $200 off) rather than percentage terms (e.g., 10% off) increases the perceived value of savings (12) by ensuring that small percentage benefits are not overlooked due to mental accounting, which leads people to focus on the relative size of savings in a spending category instead of the absolute savings (11). Additionally, people can struggle with interpreting numbers presented in unfamiliar formats (9), and interpreting percentages is especially challenging for those who are low in numeracy (10, 23). As a result, expressing savings in percentage terms can increase the amount of cognitive effort required for information processing (24), which could, in theory, lead people to perceive smaller numerical differences in the benefits that are calculated (25) and reduce decision quality for individuals who are low in numeracy (10). Consistent with these accounts, a recent field study found that describing retirement savings in dollar terms rather than percentage terms increased savings adoption among low-income respondents (26). However, more recent laboratory research found that describing large differences between values in percentage terms (e.g., 500% in earnings) can increase their perceived magnitude compared to describing them in dollar terms (e.g., $500 in earnings), because people typically evaluate percentages on a scale from 0 to 100 (27), so numbers close to or over 100 can seem to approach or exceed the scale maximum. It is therefore an open question whether describing the potential savings a borrower could achieve from signing up for an IDR plan in dollar or percentage terms would lead to more plan signups. Our experiment aims to shed light on whether theories of numeracy and mental accounting, or theories of scale-based magnitude perceptions, better explain how people make consequential decisions in the field.

Second, we examine whether the theorized benefits of consistent repetition (28) outweigh the theorized benefits of simplification (13) or vice versa when communicating with millions of borrowers. Specifically, we test whether communications encouraging borrowers to take two beneficial actions (i.e., signing up for an IDR plan and auto debit) are more impactful when they i) immediately recommend two actions in a single message with a follow-up message repeating the same two recommendations, or ii) are simplified to suggest taking one step at a time: action one (apply for IDR) in a first message, and action two (sign up for auto debit) in a second message. Research on choice overload suggests that it can be paralyzing to be presented with too many options, particularly when decisions are difficult or preferences are uncertain (29), and in general, simpler messages can improve follow-through (13, 16). On the other hand, presenting two steps in a single message allows for more repetition across multiple messages, which has also been shown to improve follow-through (17, 19, 28, 30). Again, our study addresses an open question where different theories make different predictions: when holding constant the number of communications, is it better to deliver simplified one-step-at-a-time instructions or to consistently repeat multistep instructions?

Our experiment was conducted in the fall of 2023 at the conclusion of the U.S. Department of Education’s emergency loan repayment pause (initiated at the start of the COVID-19 pandemic). The pause, a period of “mandatory administrative forbearance,” lasted from March 13, 2020, to September 1, 2023, and entailed a suspension of all required federal loan payments, the assignment of a 0% interest rate to all federal student loans, and a freeze on postdefault involuntary collections (31). Those with student debt who missed a payment immediately after this period were uniquely positioned to benefit from proactive outreach about loan repayments: after a long period of dormancy, good habits are particularly challenging to restore (32). Our intervention emails supplemented existing communications sent by Federal Student Aid, an office of the Department of Education that manages federal student financial assistance programs.

In the remainder of this paper, we present the results of our preregistered six-arm randomized controlled trial, conducted in partnership with the U.S. Department of Education. We test the impact of different messaging interventions on i) applications for IDR plans, ii) estimated auto debit payment signups, iii) on-time loan payments, and iv) estimated 60-d delinquencies.^*^

Our experimental results demonstrate that behaviorally-informed emails are effective at scale, reducing estimated delinquencies by 0.42 percentage points. We also find that reminder emails (which follow an initial message) can enhance the efficacy of communications designed to improve borrower outcomes. Further, we present the results of two preregistered tests of competing theories regarding what components of behaviorally-informed messages make them most effective. First, we find that framing potential financial savings in percentage terms is more effective than describing savings in dollar terms. Second, we find that repeatedly encouraging borrowers to sign up for both IDR plans and auto debit (in every message sent) yields better results than simplifying messages to encourage a single action at a time.

## Methods

**Fig. 1.**
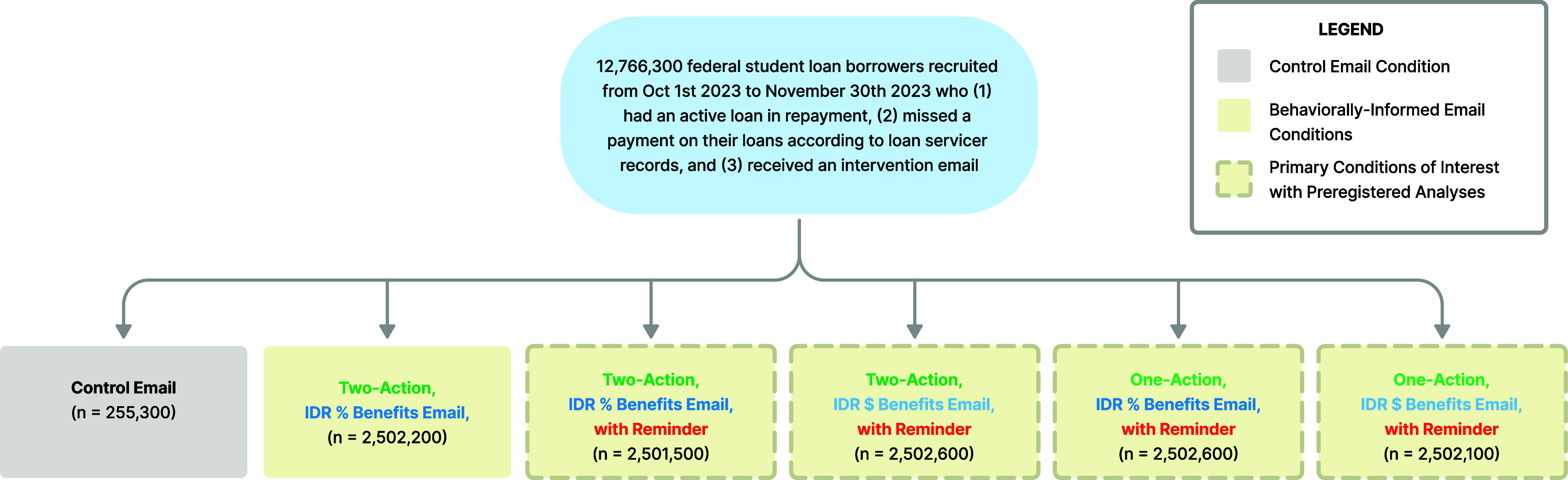
CONSORT Diagram. All sample sizes reported above are rounded to the nearest hundred borrowers in accordance with disclosure policies required by the Department of Education.

In the fall of 2023, the U.S. Department of Education sent sets of intervention emails that we collaboratively developed to federal student loan borrowers who met the following eligibility criteria: i) they had at least one active federal student loan in repayment and ii) they had missed a payment on at least one of their student loans following the end of the federal loan repayment pause on September 1st, 2023. Following our study’s preregistration, student borrowers were included in our experimental analysis of the effects of these emails if they met these eligibility criteria and received an intervention email between October 1st, 2023, and November 30th, 2023 (see Fig. 1 for details of study design).^†^ In total, 12,766,300 eligible borrowers were randomly assigned to receive one of six sets of intervention emails in the month after they missed a student loan payment (according to loan servicer records).^‡^, ^§^ A control email simply informed borrowers that they may have missed a loan payment and to contact their servicer if they believed there was an error; this email emulated a standard “business as usual” message sent by loan servicers after a missed payment. All five treatment emails provided information about the potential benefits of IDR and auto debit, and encouraged borrowers to sign up for each program. Within these treatment messages, we varied whether the emails i) did vs. did not send an identical follow-up reminder after 3 d, ii) described the potential savings from signing up for an IDR plan in dollars vs. percentage terms, and iii) included both the suggestion to sign up for IDR and auto debit in the initial and follow-up email vs. only suggested IDR signup in the initial email and only auto debit signup in the follow-up email (see *SI Appendix*, Figs. S1–S6 for details on the differences between conditions).

Borrowers in the experiment maintained their treatment assignment throughout the intervention period (which concluded on May 28th, 2024), which meant they received the same type of email communication for each missed payment on a monthly basis. Borrowers in the experiment did not receive any of the six aforementioned email variations if they made an on-time payment in the prior month, but otherwise continued to receive ongoing monthly messages. All study materials were approved by the Institutional Review Board of the University of Pennsylvania, which also approved a waiver of informed consent because participation posed minimal risk to borrowers and the experiment could not be practically carried out with informed consent. Our study was preregistered on OSF (Preregistration 1: https://bit.ly/3PjAg0C; Preregistration 2: https://bit.ly/41ZBUvT; see *SI Appendix*, section 1, for additional details).

Our two primary preregistered outcomes of interest were whether student borrowers i) applied for an IDR plan and ii) signed up for auto debit payments, both within 90 d of receiving their first email in the study. Our two secondary preregistered outcomes of interest were whether student borrowers i) made at least one loan payment within 90 d of receiving their first email and ii) lapsed into 60-d delinquency (i.e., an overdue payment for 60 d or more) within 180 d of receiving their first email. Because detailed information for every loan was aggregated to the borrower level, we estimate auto debit signups and 60-d delinquencies following algorithms described in detail in *SI Appendix*, sections 3 and 4.

## Results

### Summary Statistics.

Of the 12,766,300 borrowers in our study, 62% were female, 39% had graduated from their undergraduate institution, 63% had experienced a 60-d delinquency on a federal educational loan at some point in the past, and 21% had defaulted on an educational loan at some point in the past. Student borrowers, on average, held $34,655 in total outstanding loan balances and owed $340 in loan payments each month. For more information about the characteristics of the student borrowers in our study, see *SI Appendix*, Table S1.

Confirming that randomization was successful, we observe only two imbalances across 40 balance tests, and these involve imbalances in the presence of missing values for borrower age (0.01% missing) and whether a borrower experienced a loan default in the past (0.01% missing; these missing variables are correlated at r = 0.999), a rate of imbalance that is roughly what we would expect due to chance (see *SI Appendix*, Table S1 and section 2 for more details).

### Overview of Analysis Strategy.

Our analyses are broken into four parts: i) testing the impact of behaviorally-informed emails against sending a standard “business-as-usual” email, ii) testing the impact of sending an additional reminder email, iii) testing a preregistered comparison of the effectiveness of presenting potential financial savings in percentage vs. dollar terms, and iv) testing a preregistered comparison of recommending two actions across two emails vs. a single action within each email.

Our primary analyses rely on ordinary least squares (OLS) regressions to estimate differences in our dependent variables of interest as a function of borrowers’ experimental condition assignment. All analyses include preregistered controls for borrower demographics (age, gender, state, highest education level, and graduation from most recent educational institution); past borrower behavior^¶^ (previous 60-d delinquency, previous loan default, auto debit enrollment status in February 2020, IDR enrollment status in February 2020, loan repayment type in March 2020, and percentage of months with a payment that was overdue for 30 d or more between January 2015 and March 2020); loan attributes (total funds obtained from federal student loans, current outstanding loan balance on all loans, total monthly payments owed, days since taking out last federal student loan, loan type indicators, and loan servicer indicators); and cohort-specific fixed effects (for the borrower’s first date of a missed loan payment, which triggered the receipt of their first email in the study). Our key results are all robust to replacing our OLS model with a logistic regression model (*SI Appendix*, section 8 and Tables S4–S6).

### Benefits of Behaviorally-Informed Emails and Follow-Up Reminder Messages.

**Fig. 2.**
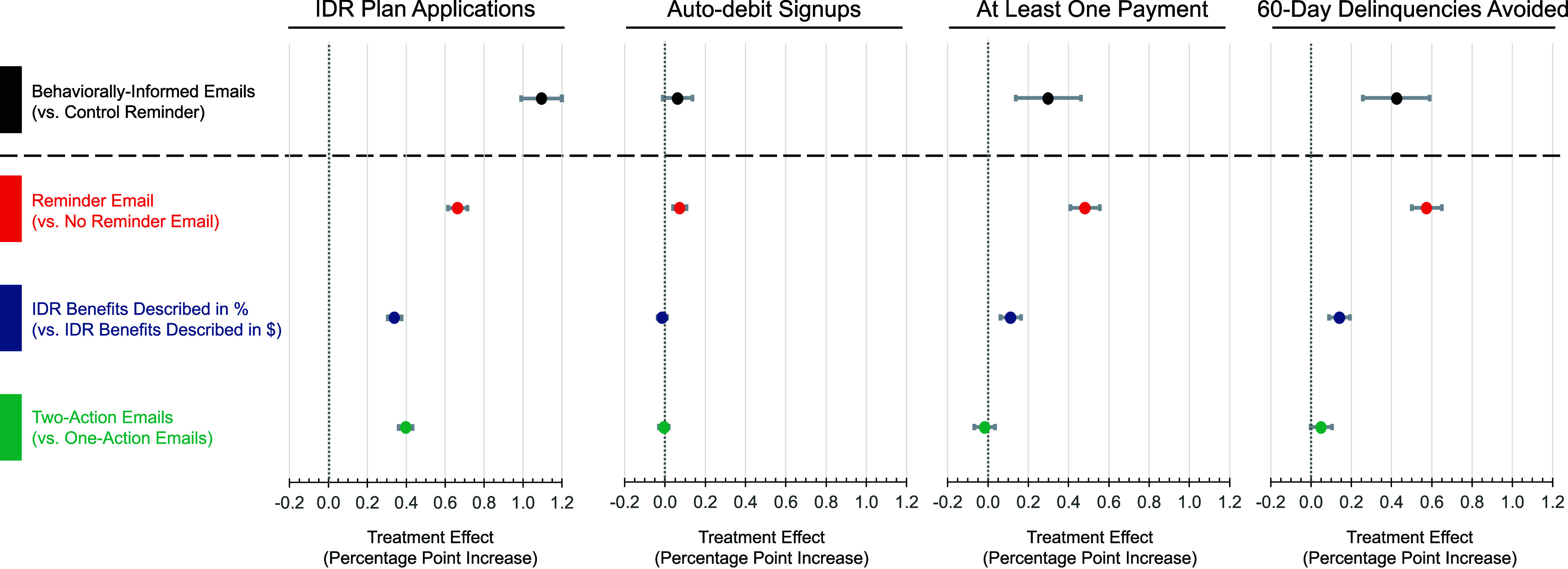
Regression-estimated percentage point improvements in borrower outcomes achieved by i) sending behaviorally-informed emails, ii) sending a reminder email, iii) describing IDR benefits in percentage terms (vs. dollar terms), and iv) sending two-action emails (vs. one action emails). Dots represent the regression-estimated percentage point improvement in the outcome of interest, while error bars represent 95% CI (based on treatment effects estimated in Tables 1–3).

First, we tested whether the pooled set of behaviorally-informed email variations outperformed the control email condition. As shown in Fig. 2 and Table 1, Models 1 to 4, behaviorally-informed emails: i) increased IDR applications by a regression-estimated 1.09 percentage points (*P* < 0.001; see Model 1; a 16.91% increase^#^), ii) had no significant impact on auto debit signups (the regression-estimated change was 0.06 percentage points; *P* = 0.102; see Model 2), iii) increased on-time payments by a regression-estimated 0.30 percentage points (*P* < 0.001; see Model 3; a 1.00% increase), and iv) reduced 60-d delinquencies by a regression-estimated 0.42 percentage points (*P* < 0.001; see Model 4; a 0.60% decrease).

**Table 1.** Regression-estimated impact of the pooled set of behaviorally-informed emails on whether a borrower applied for an IDR plan within 90 d of receiving an intervention email (Model 1), signed up for auto debit within 90 d of receiving an intervention email (Model 2), made at least one loan payment within 90 d of receiving an intervention email (Model 3), and entered into 60-d delinquency within 180 d of receiving an intervention email (Model 4)

	Model 1	Model 2	Model 3	Model 4
Behaviorally-informed emails	0.01095***	0.00062	0.00299***	−0.00424***
	(0.00053)	(0.00038)	(0.00083)	(0.00085)
Are controls included?	Yes	Yes	Yes	Yes
Number of observations	10,768,500	12,495,400	12,766,300	12,766,300
Adjusted R-squared	0.02	0.05	0.18	0.13

***P < 0.001, **P < 0.01, *P < 0.05, ^+^*P* < 0.10

*Notes:* This table reports the results of four OLS regressions including all borrowers in our experiment. The dependent variables are a binary indicator for whether a borrower applied for an IDR plan within 90 d of first receiving an intervention email (Model 1), a binary indicator for whether a borrower signed up for auto debit within 90 d of first receiving an intervention email (Model 2), a binary indicator for whether a borrower made at least one loan payment within 90 d of first receiving an intervention email (Model 3, and a binary indicator for whether a borrower lapsed into 60-d delinquency within 180 d of first receiving an intervention email. The primary predictor variable is an indicator for assignment to a condition in which a borrower received a behaviorally-informed email (with the control condition as the comparison group). For all models, control variables were included for borrower age as of August 2023, an indicator for whether the borrower was male, indicators for education grade level reported on the borrower’s last FAFSA application as of August 2023, an indicator for whether the borrower graduated from their last reported educational institution attended as of August 2023, indicators for the borrower’s state of residence as of August 2023, the number of days that elapsed between when a borrower took out their last loan as of August 2023 and when they first received an intervention email, the total funds loaned to the borrower as of August 2023, the borrower’s current outstanding loan balance as of August 2023, the borrower’s monthly loan payment amount owed as of August 2023, an indicator for a borrower’s loan servicer as of August 2023, an indicator for the borrower’s last loan repayment type prior to the repayment pause as of March 2020 (e.g., standard repayment plan, graduated repayment plan, etc.), indicators for each loan type owned by borrower as of August 2023 (i.e., Parent PLUS loans, consolidation loans), an indicator for ever having a loan payment past due for 60 d or more (60-d delinquency) as of August 2023, an indicator for ever having a loan in default as of August 2023, an indicator for whether the borrower was enrolled in an IDR (income-driven repayment) plan in February 2020 (prior to the repayment pause), an indicator for whether the borrower was signed up for auto debit in February 2020 (prior to the repayment pause), the % of months the borrower ever had a loan payment past due for 30 d or more (30-d delinquency) from January 2015 to March 2020 (prior to the repayment pause), and indicators for whether the participants first missed a payment on all possible missed payment dates (prior to eligibility for study participation being determined and prior to receiving an intervention treatment email). If a control variable contained missing data, we addressed it in two steps: i) for binary or categorical variables, missing values were replaced with zero; for continuous variables, missing values were replaced with the mean that variable took on in nonmissing observations; ii) we added an indicator variable to the regression, coded as one if the original variable was missing and zero otherwise. For Model 1, borrowers who had already signed up for a Saving on a Valuable Education (SAVE) plan as of the send date of our first intervention email message and borrowers who were ineligible to apply for a SAVE plan were excluded from our analysis. For Model 2, borrowers who had signed up for auto debit on all their loans as of the send date of our first intervention email message were excluded from our analysis. All SE shown are HC1 robust SE. All sample sizes reported above are rounded to the nearest hundred borrowers in accordance with disclosure policies required by the Department of Education.

As shown in Fig. 2 and Table 2, Models 1 to 4, sending an additional follow-up reminder email to borrowers after a missed payment i) increased IDR applications by a regression-estimated 0.66 percentage points (*P* < 0.001; see Model 1; a 9.02% increase compared to borrowers who were assigned to receive the same message but only via a single email^#^), ii) increased auto debit signups by a regression-estimated 0.07 percentage points (*P* < 0.001; see Model 2; a 1.92% increase), iii) increased on-time payments by a regression-estimated 0.48 percentage points (*P* < 0.001; see Model 3; a 1.61% increase), and iv) reduced 60-d delinquencies by a regression-estimated 0.57 percentage points (*P* < 0.001; see Model 4; a 0.82% decrease).

**Table 2.** Regression-estimated impact of sending a reminder email on whether a borrower applied for an IDR plan within 90 d of receiving an intervention email (Model 1), signed up for auto debit within 90 d of receiving an intervention email (Model 2), made at least one loan payment within 90 d of receiving an intervention email (Model 3), and entered into 60-d delinquency within 180 d of receiving an intervention email (Model 4)

	Model 1	Model 2	Model 3	Model 4
Reminder email	0.00664***	0.00073***	0.00482***	−0.00575***
	(0.00026)	(0.00017)	(0.00037)	(0.00038)
Are controls included?	Yes	Yes	Yes	Yes
Number of observations	4,220,300	4,897,600	5,003,700	5,003,700
Adjusted R-squared	0.02	0.05	0.18	0.13

***P < 0.001, **P < 0.01, *P < 0.05, ^+^*P* < 0.10

*Notes*: This table reports the results of four OLS regressions including the subset of borrowers assigned to one of two conditions in our experiment: the “Two-Action, IDR % Benefits Email with Reminder” condition or the “Two-Action, IDR % Benefits Email” condition. The dependent variables are a binary indicator for whether a borrower applied for an IDR plan within 90 d of first receiving an intervention email (Model 1), a binary indicator for whether a borrower signed up for auto debit within 90 d of first receiving an intervention email (Model 2), a binary indicator for whether a borrower made at least one loan payment within 90 d of first receiving an intervention email (Model 3), and a binary indicator for whether a borrower lapsed into 60-d delinquency within 180 d of first receiving an intervention email. The primary predictor variable is an indicator for assignment to the Two-Action, IDR % Benefits Email with Reminder condition. For all models, control variables were included for borrower age as of August 2023, an indicator for whether the borrower was male, indicators for education grade level reported on the borrower’s last FAFSA application as of August 2023, an indicator for whether the borrower graduated from their last reported educational institution attended as of August 2023, indicators for the borrower’s state of residence as of August 2023, the number of days that elapsed between when a borrower took out their last loan as of August 2023 and when they first received an intervention email, the total funds loaned to the borrower as of August 2023, the borrower’s current outstanding loan balance as of August 2023, the borrower’s monthly loan payment amount owed as of August 2023, an indicator for a borrower’s loan servicer as of August 2023, an indicator for the borrower’s last loan repayment type prior to the repayment pause as of March 2020 (e.g., standard repayment plan, graduated repayment plan, etc.), indicators for each loan type owned by borrower as of August 2023 (i.e., Parent PLUS loans, consolidation loans), an indicator for ever having a loan payment past due for 60 d or more (60-d delinquency) as of August 2023, an indicator for ever having a loan in default as of August 2023, an indicator for whether the borrower was enrolled in an IDR (income-driven repayment) plan in February 2020 (prior to the repayment pause), an indicator for whether the borrower was signed up for auto debit in February 2020 (prior to the repayment pause), the % of months the borrower ever had a loan payment past due for 30 d or more (30-d delinquency) from January 2015 to March 2020 (prior to the repayment pause), and indicators for whether the participants first missed a payment on all possible missed payment dates (prior to eligibility for study participation being determined and prior to receiving an intervention treatment email). If a control variable contained missing data, we addressed it in two steps: i) for binary or categorical variables, missing values were replaced with zero; for continuous variables, missing values were replaced with the mean that variable took on in nonmissing observations; ii) we added an indicator variable to the regression, coded as one if the original variable was missing and zero otherwise. For Model 1, borrowers who had already signed up for a SAVE plan as of the send date of our first intervention email message and borrowers who were ineligible to apply for a SAVE plan were excluded from our analysis. For Model 2, borrowers who had signed up for auto debit on all their loans as of the send date of our first intervention email message were excluded from our analysis. All SE shown are HC1 robust SE. All sample sizes reported above are rounded to the nearest hundred borrowers in accordance with disclosure policies required by the Department of Education.

### Exploration of Primary Hypotheses.

We now turn to testing the first of our two primary, preregistered research questions: whether describing the benefits of signing up for an income-driven repayment plan in terms of an absolute dollar or percentage reduction in monthly payments yields greater benefits. As shown in Fig. 2 and Table 3, Models 1 to 4, describing IDR benefits in terms of expected percentage savings on future payments (rather than absolute dollars a borrower might expect to save) was generally superior. This framing change i) increased IDR signups by a regression-estimated 0.34 percentage points (a 4.55% increase compared to borrowers who were assigned to emails describing IDR benefits in dollar terms^#^; *P* < 0.001 see Model 1), ii) produced no significant change in rates of auto debit signup (the regression-estimated change was −0.01 percentage points; *P* = 0.271, Model 2), iii) increased the percentage of borrowers who made at least one on-time payment by 0.11 percentage points (*P* < 0.001; see Model 3; a 0.37% increase), and iv) decreased the rates of 60-d delinquency by 0.14 percentage points (*P* < 0.001, Model 4; a 0.20% decrease).

**Table 3.** Regression-estimated impact of describing IDR benefits in % (vs. $) terms and recommending two recommended actions repeatedly (vs. one of two recommended action sequentially) on whether a borrower applied for an IDR plan within 90 d of receiving an intervention email (Model 1), signed up for auto debit within 90 d of receiving an intervention email (Model 2), made at least one loan payment within 90 d of receiving an intervention email (Model 3), and entered into 60-d delinquency within 180 d of receiving an intervention email (Model 4)

	Model 1	Model 2	Model 3	Model 4
IDR benefits described in %	0.00339***	-0.00013	0.00113***	−0.00141***
	(0.00018)	(0.00012)	(0.00026)	(0.00027)
Two-action email	0.00398***	−0.00006	−0.00017	−0.00051^+^
	(0.00018)	(0.00012)	(0.00026)	(0.00027)
Are controls included?	Yes	Yes	Yes	Yes
Number of observations	8,442,700	9,796,300	10,008,800	10,008,800
Adjusted R-squared	0.02	0.05	0.18	0.13

***P < 0.001, **P < 0.01, *P < 0.05, ^+^*P* < 0.10

*Notes*: This table reports the results of four OLS regressions including the subset of borrowers assigned to one of four conditions in our experiment: the “Two-Action, IDR % Benefits Email with Reminder” condition, the “Two-Action, IDR $ Benefits Email with Reminder” condition, the “One-Action, IDR % Benefits Email with Reminder” condition and the “One-Action, IDR $ Benefits Email with Reminder” condition. The dependent variables are a binary indicator for whether a borrower applied for an IDR plan within 90 d of first receiving an intervention email (Model 1), a binary indicator for whether a borrower signed up for auto debit within 90 d of first receiving an intervention email (Model 2), a binary indicator for whether a borrower made at least one loan payment within 90 d of first receiving an intervention email (Model 3), and a binary indicator for whether a borrower lapsed into 60-d delinquency within 180 d of first receiving an intervention email. The primary predictor variables are indicators for whether a borrower received communications that described IDR benefits in % (vs. $) terms and whether a borrower received communications that recommended two actions repeatedly (vs. communications that recommended one of two recommended actions sequentially). For all models, control variables were included for borrower age as of August 2023, an indicator for whether the borrower was male, indicators for education grade level reported on the borrower’s last FAFSA application as of August 2023, an indicator for whether the borrower graduated from their last reported educational institution attended as of August 2023, indicators for the borrower’s state of residence as of August 2023, the number of days that elapsed between when a borrower took out their last loan as of August 2023 and when they first received an intervention email, the total funds loaned to the borrower as of August 2023, the borrower’s current outstanding loan balance as of August 2023, the borrower’s monthly loan payment amount owed as of August 2023, an indicator for a borrower’s loan servicer as of August 2023, an indicator for the borrower’s last loan repayment type prior to the repayment pause as of March 2020 (e.g., standard repayment plan, graduated repayment plan, etc.), indicators for each loan type owned by borrower as of August 2023 (i.e., Parent PLUS loans, consolidation loans), an indicator for ever having a loan payment past due for 60 d or more (60-d delinquency) as of August 2023, an indicator for ever having a loan in default as of August 2023, an indicator for whether the borrower was enrolled in an IDR (income-driven repayment) plan in February 2020 (prior to the repayment pause), an indicator for whether the borrower was signed up for auto debit in February 2020 (prior to the repayment pause), the % of months the borrower ever had a loan payment past due for 30 d or more (30-d delinquency) from January 2015 to March 2020 (prior to the repayment pause), and indicators for whether the participants first missed a payment on all possible missed payment dates (prior to eligibility for study participation being determined and prior to receiving an intervention treatment email). If a control variable contained missing data, we addressed it in two steps: i) for binary or categorical variables, missing values were replaced with zero; for continuous variables, missing values were replaced with the mean that variable took on in nonmissing observations; ii) we added an indicator variable to the regression, coded as one if the original variable was missing and zero otherwise. For Model 1, borrowers who had already signed up for a SAVE plan as of the send date of our first intervention email message and borrowers who were ineligible to apply for a SAVE plan were excluded from our analysis. For Model 2, borrowers who had signed up for auto debit on all their loans as of the send date of our first intervention email message were excluded from our analysis. All SE shown are HC1 robust SE. All sample sizes reported above are rounded to the nearest hundred borrowers in accordance with disclosure policies required by the Department of Education.

Turning to our second primary, preregistered research question, we explore whether it is more effective to send borrowers messages that recommend two actions pertaining to loan repayments in one email (the first action encouraged was to sign up for IDR, and the second action encouraged was to sign up for auto debit) or to separately encourage these two actions across two separate emails (the first email encouraging sign up for IDR, and the second encouraging sign up for auto debit). As shown in Fig. 2 and Table 3, Models 1 to 4, compared with emails suggesting one action at a time, emails suggesting two actions at a time i) increased IDR application rates by a regression-estimated 0.40 percentage points (*P* < 0.001; see Model 1; a 5.35% increase relative to borrowers who were assigned to receive one action at-a-time emails^#^), ii) had no significant impact on auto debit signups (the regression-estimated change was 0.01 percentage points; *P* = 0.617; see Model 2), iii) had no significant impact on whether a borrower made at least one, on-time payment (the regression-estimated change was −0.02 percentage points; *P* = 0.530, see Model 3), and iv) decreased 60-d delinquencies by a marginally significant 0.05 percentage points (*P* = 0.058; see Model 4).

### Are the Benefits of Two Action Emails Generalizable?

There is a risk that our findings regarding the benefits of two action emails (over one action emails) could be driven by the specific two actions we recommend in this study or by the order in which those actions were presented (namely: IDR plan signup and auto debit signup, with IDR plan signup presented first). To address this issue, we abstract away from the policy outcomes of interest and look only at whether two-action emails changed recipients’ actions (i.e., clicking links) more than one action emails.

If we abstract away from this case, we might assume a policy maker has two actions they seek to promote among constituents, and that each action, if completed, would generate equal benefit to those constituents. In this scenario, the goal for policy makers would be to maximize overall action-taking, and policy makers would accept some level of substitution between actions.

Analyzing our data with this in mind, we examine whether two-action emails generated more people clicking on links to take recommended action steps than one-action emails.^||^ As shown in *SI Appendix*, Table S2, Model 3, we find that sending two, two-action emails increased the total number of people clicking links by 0.21 percentage points compared to sending two, one-action emails (*P* < 0.001). We also observe modest substitution between the types of links that recipients click across conditions. As shown in *SI Appendix*, Table S2, Model 1, the two, two-action emails increased the number of people clinking on the (first) link encouraging IDR plan applications by 1.44 percentage points (*P* < 0.001). But as shown in Model 2, these same emails decreased the number of people clicking on links facilitating auto debit signup by 1.30 percentage points (*P* < 0.001; auto debit signup was the second recommended action step across emails). When we use seemingly unrelated estimation to compare the regression-estimated effects of the two, two-action emails across these two models (*SI Appendix*, Table S2, Models 1 and 2), we find that the estimated increase in people clicking the IDR application link is significantly greater than the estimated decrease in people clicking the auto debit signup (or “contact your loan servicer”) link (*P* < 0.001).

In our case (as in our generalized scenario), it is ambiguous which action (IDR or auto debit signup) is more beneficial for recipients; we therefore view an increase in total clicks in the two-action email condition as providing support for the value of this outreach approach more generally.

### Exploration of Heterogeneous Treatment Effects.

To explore whether our interventions generated heterogeneous treatment effects, we focus on the most proximal and malleable outcome variable measured: applications for IDR plans (our sample size gave us 99% power to detect a minimum effect size of 0.21 percentage points at a 0.05 significance level for this outcome). We find fairly consistent evidence of heterogeneous treatment effects on this outcome.

First, we note that several subpopulations consistently showed greater treatment effects on IDR applications across all of our tested interventions (behaviorally-informed emails, reminder emails, framing IDR benefits in percentage terms, and encouraging two actions repeatedly across two emails). These subpopulations include i) women (on average, treatment effects were 47 to 59% larger for women than men), ii) borrowers who were enrolled in an IDR plan prior to the repayment pause (on average, treatment effects were 41 to 87% larger for borrowers who were enrolled in IDR prior to the repayment pause than for borrowers who were not), iii) borrowers with a higher outstanding loan balance (on average, treatment effects were 3 to 4% larger for borrowers with an additional $10,000 in outstanding loan balances), and iv) borrowers who had received a more recent loan disbursement (on average, treatment effects were 3 to 4% larger for each year’s reduction in years since a borrower’s most recent loan disbursement; see *SI Appendix*, Table S3 and section 10 for more details).

We also observe that borrowers who were not enrolled in auto debit before the repayment pause and borrowers not on a standard repayment plan benefited more from behaviorally-informed emails than others, and that reminder messages had larger benefits for older borrowers, borrowers with a higher monthly payment, borrowers who received more funds from student loans, borrowers who had at least one consolidation loan, borrowers who were not enrolled in auto debit before the repayment pause, and borrowers not on a standard repayment plan. Framing IDR benefits in percentage terms was also more effective for borrowers who received more funds from student loans, borrowers who did not graduate from their last educational institution, and borrowers who were not enrolled in auto debit before the repayment pause. Finally, two-action messages also had bigger benefits for older borrowers, borrowers with a higher monthly payment, borrowers who received more funds from student loans, borrowers with at least one consolidation loan, and borrowers who were not on a standard repayment plan. While these analyses were exploratory, 17% of the 320 two-tailed statistical tests we analyzed suggested significance at the 5% level, suggesting it is unlikely all of the detected heterogeneity is purely noise, but confirmatory follow-up studies to explore this intriguing heterogeneity would be valuable.

## Discussion

This preregistered experiment involving 12,766,300 student borrowers demonstrates that timely, behaviorally-informed emails can help improve repayment outcomes. Our paper also highlights the effectiveness of communicating potential financial savings in percentage terms, and emphasizes the value of repeated reminders in communications emphasizing more than one action at a time. This latter result extends past research on the benefits of sending reminders to supplement an initial communication (17, 19, 30).

Our experiment provides evidence addressing two research questions. First, we provide evidence in a consequential field setting that describing the financial benefits (i.e., savings) a person could obtain in percentage terms is more persuasive (producing 4.5% more IDR sign-ups) than framing those same benefits in dollar terms. This finding aligns with a recent laboratory study by Klusowski and Lewis (27) showing that describing large financial gains in percentage terms (rather than dollar terms) can amplify the perceived magnitude of a benefit. Notably, our finding stands in contrast to prior laboratory research, which suggests that framing savings in dollar terms could enhance the perceived magnitude of savings either because dollars are more familiar and interpretable (9) or because emphasizing large dollar amounts can be an effective antidote to people’s tendency to diminish the perceived magnitude of savings in large mental accounts (11, 12).

Second, although past research has highlighted the benefits of simplification [see Sunstein (13) for a review], we show that two emails that consistently repeated the same two actions were more effective than two separate emails, each suggesting one action. We show a two-action email in our study generated more total clicks on both recommended actions than emails encouraging one action at a time, with the total click increase offsetting a modest substitution effect, through which the two-action email attracted more people to click on the first recommended action at the cost of clicks for the second recommended action. These findings highlight the value of both i) suggesting two actions repeatedly (rather than suggesting one action at a time separately), but also of ii) presenting the most valuable action first in scenarios where policy makers have evidence to suggest one action is more beneficial to constituents than the other. Notably, the results of simplification could be very different in a case where there are three or more recommended action steps, but our findings suggest that two action steps need not be separated, and the returns from repeatedly messaging people about two recommendations exceed the cost of added complexity from placing them in a single message.

Our findings have a number of important practical implications. If our best-performing email were scaled to all 13-million borrowers in our experimental sample, we estimate that our behaviorally-informed messages would have prompted 183,500 to 211,300 additional borrowers to apply for an IDR plan, encouraged 34,300 to 77,400 additional borrowers to make at least one loan payment, and prevented 57,700 to 101,900 additional borrowers from lapsing into 60-d delinquency (ranges represent 95% CI; for details, see *SI Appendix*, section 11). These benefits can be achieved at nearly zero marginal cost (33). Our interventions were consistently effective across the diverse population of student loan borrowers in the United States, although borrowers who were women, had a higher loan balance, had a more recent loan, and had previously signed up for an IDR plan prior to the repayment pause particularly benefited.

Our study has several important limitations. First, our experiment was conducted at one (arguably unique) point in time: right after the COVID-19 student loan repayment pause ended in September 2023, which involved an “on-ramp” that limited the penalties associated with delinquencies, and after the rollout of a new IDR plan. It is difficult to determine whether the measured effects might be larger or smaller in magnitude had the experiment been conducted several years earlier or later. Second, all of our messages encouraged borrowers to sign up for an IDR plan before encouraging them to sign-up for auto debit payments. Ideally, we would have randomized the order in which these actions were encouraged, but we were unable to do so in this study. Finally, our field experiment focused only on changing the repayment behaviors of U.S. student borrowers, and we might have obtained different results had we focused on changing other policy-relevant behaviors (e.g., commercial loan repayments or the adoption of SNAP benefits). Future research testing the same theories on a wider range of outcome variables would be valuable.

Overall, our experiment shows that low-cost nudges (33) are a cost-effective policy tool for generating scalable improvements in the loan repayment and delinquency rates of millions of American student loan borrowers (34). While system-level changes may be needed to address student loan repayment difficulties (35), the interventions tested here are designed to complement more costly approaches to improving the student loan repayment system. Our findings reinforce past research suggesting that repeated reminder messages are often valuable. They also suggest that emphasizing two steps a person can take to improve their outcomes and framing expected savings benefits in percentage terms can create value. These same principles may be useful for nudging other beneficial actions such as encouraging the repayment of commercial loans and the adoption of other beneficial government programs.

## Supplementary Material

Appendix 01 (PDF)

## Data Availability

The data analyzed in this paper were provided by the Department of Education. We cannot publicly post individual-level data on student debt or repayment decisions that we received from the Department of Education because it is protected financial data owned by the U.S. Government. Government data containing individual-level financial information is not made publicly available to protect privacy (as even if the data are de-identified, it is still possible to re-identify people from de-identified data). However, data and code used in this paper will be maintained by the Office of the Chief Economist in consideration of potential replication requests. Katherine L. Milkman (kmilkman@wharton.edu) and Benjamin L. Castleman (castleman@virginia.edu) should be contacted by interested researchers to facilitate requests for data sharing via the Office of the Chief Economist.

## Footnotes

^*^The structure of our dataset required us to estimate auto debit signups and delinquencies; we discuss how we do this in *SI Appendix*, sections 3 and 4.

^†^Access to the data analyzed in this paper was restricted to researchers working for the U.S. Department of Education due to its classification as protected financial data owned by the federal government. To safeguard privacy, individual-level data is not publicly available. At the time of the study, Robert Kuan was an intern at the Department and Kristin Blagg was a full-time employee, which allowed Robert and Kristin to execute the data analysis. At the start of the study, Jordan Matsudaira was the Department’s Chief Economist and Lesley Turner was an expert at the Department, and both supervised the research as well as the execution of the experiment.

^‡^All borrowers were randomly assigned to treatments using a borrower ID that contained a sequence of random, uniformly distributed digits. Ranges of the ID sequence were then used to divide borrowers into predetermined treatment groups.

^§^The U.S. Department of Education relies on private companies (“loan servicers”) to manage federally-owned student loans through contracts. Servicers typically provide bills, collect payments, and handle delinquencies. Servicers also regularly report data to the U.S. Department of Education on loan status and payment activity for the borrower.

^¶^While the majority of our controls are as of August 2023 (before the start of our experiment), some measures of past borrower behavior are as of or before March 2020 (when the COVID-19 repayment pause took effect).

^#^See *SI Appendix*, section 7 for details on how we calculate all reported percentage increases, which require computing the baseline rates of i) IDR applications, ii) auto debit signup, iii) on-time loan payment, and iv) 60-d delinquency.

^||^Links at the bottom of all emails invited borrowers to “contact your loan servicer” (if they needed assistance) and took them to the same webpage as links inviting them to sign up for auto debit, so we simultaneously track interest in auto debit signup and servicer help-seeking in these analyses.
