# Supplementary material for: Behavioral nudges prevent loan delinquencies at scale: A 13-million-person field experiment
Source: Proc Natl Acad Sci U S A. 2025 Jan 23;122(4):e2416708122. doi: 10.1073/pnas.2416708122 (PMC11789030; doi:10.1073/pnas.2416708122)
Supplement: Supplementary file 1 — Appendix 01 (PDF) [file pnas.2416708122.sapp.pdf]

PNAS

Supporting Information for

Behavioral nudges prevent loan delinquencies at scale: a 13-million-person field experiment

Robert Kuan[a,1], Kristin Blagg[b], Benjamin L. Castleman[c], Rajeev Darolia[d,e], Jordan D. Matsudaira[f], Katherine L. Milkman[a,1], Lesley J. Turner[d,g]

[a] Department of Operations, Information and Decisions, The Wharton School, University of Pennsylvania; Philadelphia, PA 19104
[b] Center on Education Data and Policy, The Urban Institute; Washington, DC 20024
[c] The Frank Batten School of Leadership and Public Policy, University of Virginia; Charlottesville, VA 22904
[d] Office of the Chief Economist, U.S. Department of Education; Washington, DC 20202
[e] The James W. Martin School of Public Policy and Administration, University of Kentucky; Lexington, KY 40506
[f] Department of Public Administration and Policy, The School of Public Affairs, American University; Washington, DC 20016
[g] The Harris School of Public Policy, University of Chicago; Chicago, IL 60637

[1] To whom correspondence may be addressed: Robert Kuan (email: rkuan@wharton.upenn.edu) and Katherine L. Milkman (email: kmilkman@wharton.upenn.edu)

This PDF file includes:

Supporting text
Figures S1 to S6
Tables S1 to S8

Table of Contents

1. Preregistration and Recruiting Target 3

2. How Balance Checks Were Conducted 3

3. How We Calculated Our Proxy Measure for Whether Borrowers Signed Up For Auto Debit in the 90 Days Post-Intervention 3

4. How We Calculated Our Proxy Measure for Whether Borrowers Lapsed Into 60-Day Delinquency in the 180 Days Post-Intervention 4

5. Known Data Imperfections 5

6. Details on the Dataset and Assumptions Used in Our Link Click Analysis 5

7. Estimating Baseline Rates of IDR Signup, Auto debit Signup, On-Time Loan Payments and 60-Day Delinquency 5

8. Robustness to Logit Regression Modeling 6

9. Treatment Effects after 30 Days and 60 Days 6

10. Heterogeneity Analysis 6

11. Estimates of the Incremental Borrowers Affected If Our Best Performing Intervention Email Was Used 6

Figures 8

Tables 14

Supporting Information Text

1. Preregistration and Recruiting Target

We posted an initial pre-registration on October 23rd, 2023 (https://bit.ly/3PjAg0C), and we updated our pre-registration on October 26th, 2023 (https://bit.ly/41ZBUvT). The updated pre-registration clarified the control variables in our regression and our exclusion criteria for the study. Both pre-registrations were submitted before our experiment launched on October 27th, 2023.

In our final pre-registration, we stated that we aimed to recruit more than 12.5 million borrowers by November 30th, 2023 or to extend our participant recruitment to January 31st, 2024 if we did not reach our target sample size. From October 1st, 2023 to November 30th, 2023, we recruited 12,766,300 borrowers who (1) had an active federal loan in repayment, (2) had missed a payment on a loan according to loan servicer records, and (3) had received a treatment email, and therefore met our recruiting target by November 30th, 2023.

In our pre-registration, we specified that our OLS regression would include indicators for IDR benefits described in $ terms (vs. % terms) and for including one suggested action step in each email (vs. two). In order to increase our presentation clarity, these indicators in our analysis are replaced with indicators for IDR benefits described in % terms (vs. $ terms) and two suggested action steps in each email (vs. one). The significance, point estimates, and standard errors of our regression are identical to what they would be with the originally pre-registered versions of these indicators, except that the coefficients estimated for these indicators are reversed in sign.

2. How Balance Checks Were Conducted

To check that random assignment was successful, we conducted balance checks on 20 borrower covariates, as shown in Table S1. We regressed each of the 20 borrower covariates on indicators of assignment for each of our five treatment conditions (with the indicator for the control condition omitted) using an OLS regression with HC1 robust standard errors. Then, for each borrower covariate, we conducted an F-test to test the null hypothesis that the coefficients for all treatment conditions were equal to zero. We did not find any significant imbalance across any of the 20 borrower covariates (all p-values > 0.05).

To test for differential rates of missing values across covariates, we ran the same procedure for the balance checks described above, but we replaced the 20 borrower covariates with 20 indicators for whether each of the 20 borrower covariates took on a missing value (e.g., the borrower's age covariate was replaced with an indicator for whether the borrower's age was missing). As reported in the paper, we find two imbalances: the proportion of missing values for borrower age (0.01% missing) and whether a borrower experienced a loan default in the past (0.01% missing; these missing variables are correlated at $r = 0.999$). We do not find any significant imbalance in the proportion of missing values across the other borrower covariates (all p-values > 0.05).

For a full summary of borrower characteristics and all balance checks described above, please see Table S1.

3. How We Calculated Our Proxy Measure for Whether Borrowers Signed Up For Auto Debit in the 90 Days Post-Intervention

The exact date when a borrower signed up for auto debit was not tracked. At the time we pre-registered our analysis, we believed that we could obtain auto debit enrollment data pulls on a frequent basis and that we would be able to use enrollment data from the date of a data pull that fell closest to (i.e., at or prior to) 90 days after a participant received their first study email (see our pre-registration - https://bit.ly/41ZBUvT). However, this "snapshot" approach to capturing auto

debit signups proved infeasible. Instead, we were able to find an alternative method to calculate a more accurate proxy measure for whether borrowers signed up for auto debit: we observed whether a borrower was signed up for auto debit payments for at least one loan and whether they were signed up for auto debit on all loans at four different points in time: i) the date when a borrower received their first intervention email, (2) 30 days after this date, (3) 60 days after this date, and (4) 90 days after this date. Using this data, we construct proxy measures of whether a borrower signed up for auto debit during the 30, 60 and 90 days following enrollment in our experiment as follows. If either of the following criteria are met, we define a borrower as signed-up for auto debit within the 90 day follow-up period after the start of our intervention:

1. A borrower was not signed up for auto debit on any loans on the date of our first intervention email, but was signed up for auto debit for at least one loan 90 days after their first intervention email, or

2. A borrower was not signed up for auto debit on all of their loans on the date of our first intervention email, but was signed up for auto debit on all of their loans 90 days after their first intervention email.

To construct a proxy measure of whether a borrower signed up for auto debit in the 30 or 60 days following enrollment in our experiment, we use exactly the same procedure detailed above but replace “90 days” with an alternative time horizon (i.e., 30 days for our 30 day proxy; 60 days for our 60 day proxy).

4. How We Calculated Our Proxy Measure for Whether Borrowers Lapsed Into 60-Day Delinquency in the 180 Days Post-Intervention

We constructed our proxy measure for whether a borrower lapsed into 60-day delinquency within 180 days of receiving their first experimental email as follows. We took the maximum number of days the borrower spent in delinquency (post-October 1st and prior to the end of their intervention period) across all calculated delinquency durations, and if this maximum was 60 days or greater, we defined the borrower as having lapsed into 60-day delinquency during our study period. To calculate this maximum, we relied on a matching algorithm outlined below to pair records of dates when participants entered and exited delinquency:

1. <u>If a borrower had exited delinquency on any date within their 180 day intervention period.</u> We matched each date that the borrower exited delinquency (e.g., October 10th, 2023) with the closest prior date that the borrower was recorded as entering delinquency (e.g., October 3rd, 2023) after the loan repayment pause concluded (on September 30, 2023). We then calculated the number of days separating each matched delinquency entrance and exit (e.g., if someone entered delinquency on October 3rd, 2023, and exited delinquency on October 10th, 2023, this value would be 7).

2. <u>If a borrower had not exited delinquency on any date within their 180 day intervention period (data pulled as of June 28th, 2024)</u>. We calculated their days in delinquency by finding their closest delinquency entrance date and calculating the number of days separating it from the last date in their 180 day intervention period (e.g., if someone entered delinquency on November 1st, 2023, did not have a recorded date of exiting delinquency, and ended their 180 day intervention period on April 29th, 2024, this value would be 180).

If borrowers did not have a recorded date of entering delinquency between October 1st (when the loan repayment pause concluded) and the end of their 180-day intervention period, they were coded as not lapsing into 60-day delinquency in the 180 days post-intervention.

To construct a proxy measure of whether a borrower lapsed into delinquency in the 30 or 60 days following enrollment in our experiment, we use exactly the same procedure detailed above but

replace “180 days” with an alternative time horizon (i.e., 30 days for our 30 day proxy; 60 days for our 60 day proxy).

5. Known Data Imperfections

Our delinquency dataset contains some observations that are either infeasible (borrowers exit out of delinquency more times than they enter into delinquency) or unlikely (borrowers enter into delinquency three or more times without exiting delinquency). In our full dataset, 4.53% of observations exhibit these patterns.

Our dataset also contains borrowers who were recorded as having a missed payment prior to September 1st that triggered our first intervention email. Although the COVID-19 loan repayment pause was still in effect, a small number of borrowers opted out of the mandatory administrative forbearance and continued to make payments. In our full dataset, 0.04% of observations exhibit these patterns. We coded these missed payment dates as missing in our dataset.

6. Details on the Dataset and Assumptions Used in Our Link Click Analysis

Our data contained information on the URLs that a borrower was directed to from the initial email or set of emails sent after a borrower’s first missed loan payment. Both the auto debit call-to-action button and a link encouraging borrowers to “contact your loan servicer” directed borrowers to the same URL address, so by necessity, we track both types of link clicks together.

7. Estimating Baseline Rates of IDR Signup, Auto debit Signup, On-Time Loan Payments and 60-Day Delinquency

For our analyses estimating the impact of behaviorally-informed emails (see Table 1 in the main text) and estimating the impact of sending a follow-up email after 3 days (see Table 2 in the main text), we estimated the baseline rate for each dependent variable (IDR signup, auto debit signup, on-time loan payment, and 60-day delinquency) by re-running identical OLS regressions to those presented in Tables 1 and 2, but without including any control variables. The intercepts from these new regressions are then used to calculate our “regression-estimated baseline rates” of IDR signup, auto debit signup, on-time loan payments, and 60-day delinquency. This was not pre-registered, as we did not anticipate our need to calculate regression-estimated baseline rates of signups, payments and delinquencies.

For our analysis estimating the impact of describing IDR benefits in % (vs. $) terms, we proceed differently. Here we estimated the “dollar framing email” group mean for each of our four dependent variables by running the same four OLS regressions in Table 3, but without any controls and omitting the indicator for assignment to a condition that received two-action emails. We rely on the intercept obtained from each of these four new regressions to generate our regression-estimated baseline rates of IDR signup, auto debit signup, on-time loan payment, and 60-day delinquency. Again, this was not pre-registered due to a lack of foresight.

For our analysis estimating the impact of recommending two actions repeatedly over two emails (vs. one action at-a-time over two emails), we used a similar procedure to that described in the prior paragraph. Specifically, we estimated the “one-action email” group mean for each of our four dependent variables by running the same four OLS regressions in Table 3, but without any controls and omitting the indicator for assignment to a condition that described IDR benefits in % terms. We rely on the intercept obtained from each of these four new regressions to generate our regression-estimated baseline rates of IDR signup, auto debit signup, on-time loan payment, and 60-day delinquency. Again, this was not pre-registered due to a lack of foresight.

8. Robustness to Logit Regression Modeling

As pre-registered, we re-ran each of the twelve OLS regressions presented in our paper (in Tables 1, 2, and 3) as logistic regressions, and as shown in the supplementary materials (Models 1-4 in Table S4, Table S5, and Table S6), all results are meaningfully unchanged.

9. Treatment Effects after 30 Days and 60 Days

As we specified in the exploratory analysis section of our pre-registration, we re-ran our pre-registered regression estimating the treatment effect of describing IDR benefits in % terms (vs. dollar terms), and the treatment effect of sending two-action emails repeatedly over two emails (vs. one-action at-a-time sequentially over two emails) with several different time horizons for our key dependent variables (applying for an IDR plan, signing up for auto debit, making at least one payment, and entering into 60-day delinquency). Specifically, our primary analyses (Tables 1-3) focused on a 90-day time horizon. We re-ran our analyses to explore whether our intervention had positive effects for borrowers in as early as 30-days or 60-days after receiving the first treatment email. As shown in the supplementary materials (Models 1-4 in Table S7 and Table S8), we see significant increases in (1) borrowers applying to an IDR plan within 30 days and within 60 days of receiving a treatment email in response to our % framing and two action step treatments, (2) borrowers making at least one payment within 60 days of receiving a treatment email in response to our % framing treatment, (3) borrowers making at least one payment within 30 days and 60 days of receiving a treatment email in response to our two action step treatment, and significant decreases in (4) borrowers lapsing into 60-day delinquency within 60-days of receiving a treatment email in response to our % framing and two action step treatments.

10. Heterogeneity Analysis

Our paper presents results from twelve key regressions (see Tables 1-3), and following our pre-registration, we explored whether the treatment effects estimated in those regressions differed as a function of various borrower attributes.

Specifically, to probe for heterogeneity in our effects as a function of a given borrower attribute (e.g., borrower age), we added an interaction (or set of interactions) between the primary treatment indicator(s) in each regression (in Tables 1-3 in the main text) and a given borrower attribute of interest (e.g., age). If a borrower attribute was continuous (e.g, borrower age), we replace the original variable and its interactions with a mean-centered version of that same variable. To handle missing data, we also added an interaction (or set of interactions) between each treatment indicator in the regression and an indicator for a missing value for the borrower attribute of interest (e.g., age missing).

We explored the following possible sources of heterogeneity for 20 borrower covariates (listed in Table S1). See Table S3 for a complete list of statistically significant heterogeneous treatment effects detected in these exploratory analyses across all tested interventions and dependent variables. Because we ran so many tests, we treat all heterogeneity analyses as exploratory, as noted in our paper.

11. Estimates of the Incremental Borrowers Affected If Our Best Performing Intervention Email Was Used

In order to estimate the number of additional borrowers who would have applied to an IDR plan if our best performing intervention email had been sent to all borrowers in our experiment, we ran an OLS regression predicting whether a borrower applied to an IDR plan within 90 days of receiving our intervention, with indicators for assignment to each of our five treatment conditions (with the indicator for the control condition omitted). Our regression included the same set of controls included in the regressions in Tables 1-3 in our paper. We take the treatment coefficient

estimated for the “Two-action, IDR % Benefits Email with a Reminder” condition indicator and multiply it by the total number of borrowers in our experiment (12,766,300). This is the number of additional borrowers we assume we could have encouraged to apply for IDR plans within 90 days of treatment if the best treatment email had been deployed to all borrowers in our sample. To calculate the 95% CI surrounding this estimate, we take the upper and lower bounds on the 95% CI for the coefficient and multiply each of those quantities by the same total number of borrowers referenced above.

To estimate the number of additional borrowers who would have made at least one loan payment if our best performing intervention had been sent to all borrowers in our experiment, we conduct the same regression analysis described in the previous paragraph, but we replace the outcome with an indicator for whether a borrower made at least one student loan payment within 90 days of receiving our intervention. All follow-up calculations are done as described previously.

To estimate the number of additional borrowers who would have been prevented from entering 60-day delinquency if our best-performing intervention had been sent to all borrowers in our experiment, we conduct the same regression analysis described in the first paragraph, but we replace the outcome with an indicator for whether a borrower lapsed into 60-day delinquency. We then multiplied the coefficient on the “Two action, IDR % Benefits Email with a Reminder” condition indicator by -1 (because a negative coefficient would indicate an improvement in delinquency and an increase in the number of borrowers we prevented from lapsing into 60-day delinquency). All follow-up calculations are done as described previously.

All estimates of the number of incremental borrowers impacted are rounded to the nearest hundred borrowers in accordance with disclosure policies required by the Department of Education.

Figures

First Email	Second Email (sent 3 days later)
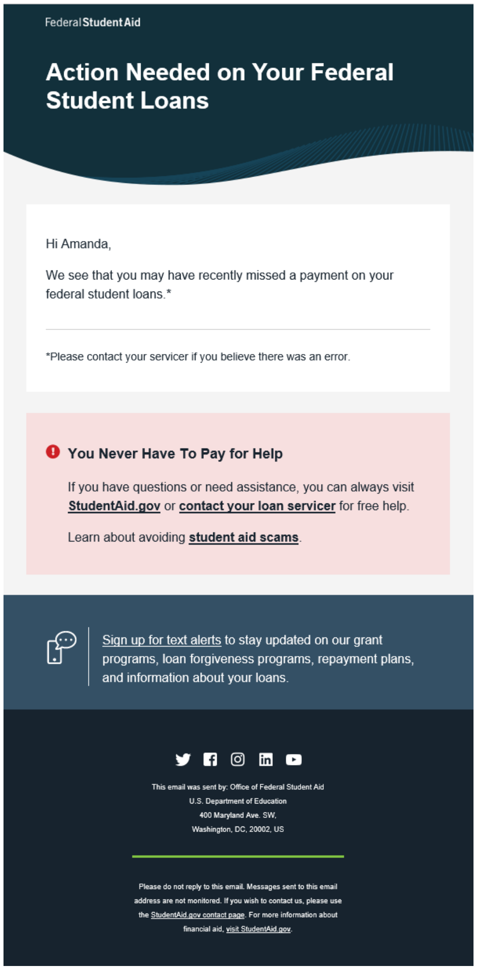	n/a

Fig. S1. Emails sent to borrowers in the control condition.

First Email	Second Email (sent 3 days later)
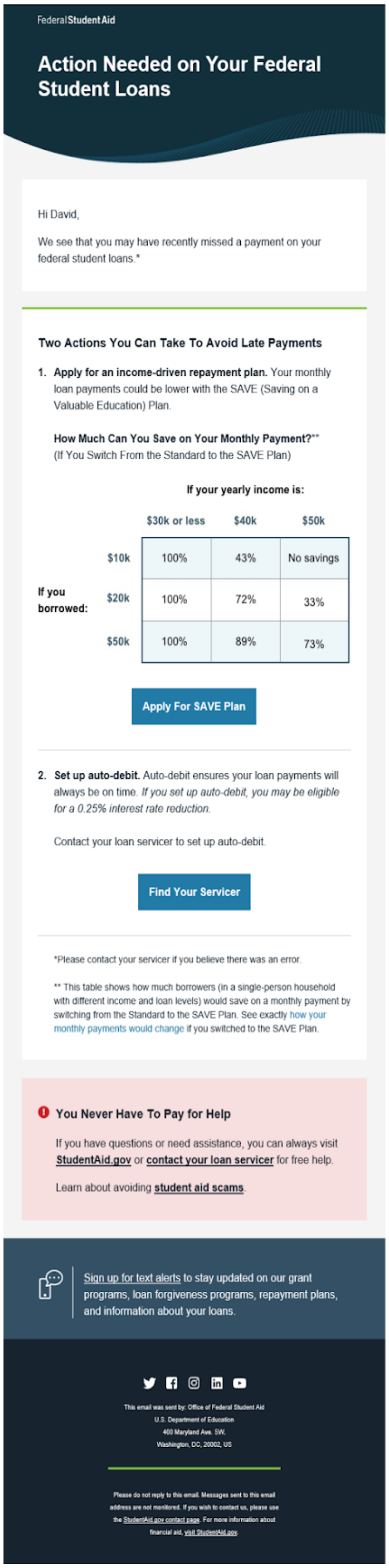	n/a

FederalStudentAid

Action Needed on Your Federal Student Loans

Hi David,

We see that you may have recently missed a payment on your federal student loans.*

Two Actions You Can Take To Avoid Late Payments

1. **Apply for an income-driven repayment plan.** Your monthly loan payments could be lower with the SAVE (Saving on a Valuable Education) Plan.

   How Much Can You Save on Your Monthly Payment?**
   (If You Switch From the Standard to the SAVE Plan)

		If your yearly income is:		
		$30k or less	$40k	$50k
If you borrowed:	$10k	100%	43%	No savings
	$20k	100%	72%	33%
	$50k	100%	89%	73%

   Apply For SAVE Plan

2. **Set up auto-debit.** Auto-debit ensures your loan payments will always be on time. *If you set up auto-debit, you may be eligible for a 0.25% interest rate reduction.*

   Contact your loan servicer to set up auto-debit.

   Find Your Servicer

*Please contact your servicer if you believe there was an error.

** This table shows how much borrowers (in a single-person household with different income and loan levels) would save on a monthly payment by switching from the Standard to the SAVE Plan. See exactly how your monthly payments would change if you switched to the SAVE Plan.

You Never Have To Pay for Help

If you have questions or need assistance, you can always visit **StudentAid.gov** or **contact your loan servicer** for free help.

Learn about avoiding **student aid scams**.

Sign up for text alerts to stay updated on our grant programs, loan forgiveness programs, repayment plans, and information about your loans.

This email was sent by: Office of Federal Student Aid
U.S. Department of Education
400 Maryland Ave. SW,
Washington, DC, 20002, US

Please do not reply to this email. Messages sent to this email address are not monitored. If you wish to contact us, please use the StudentAid.gov contact page. For more information about financial aid, visit StudentAid.gov.

Fig. S2. Emails sent to borrowers in the “two-action, IDR % benefits email without a reminder” condition.

First Email	Second Email (sent 3 days later)

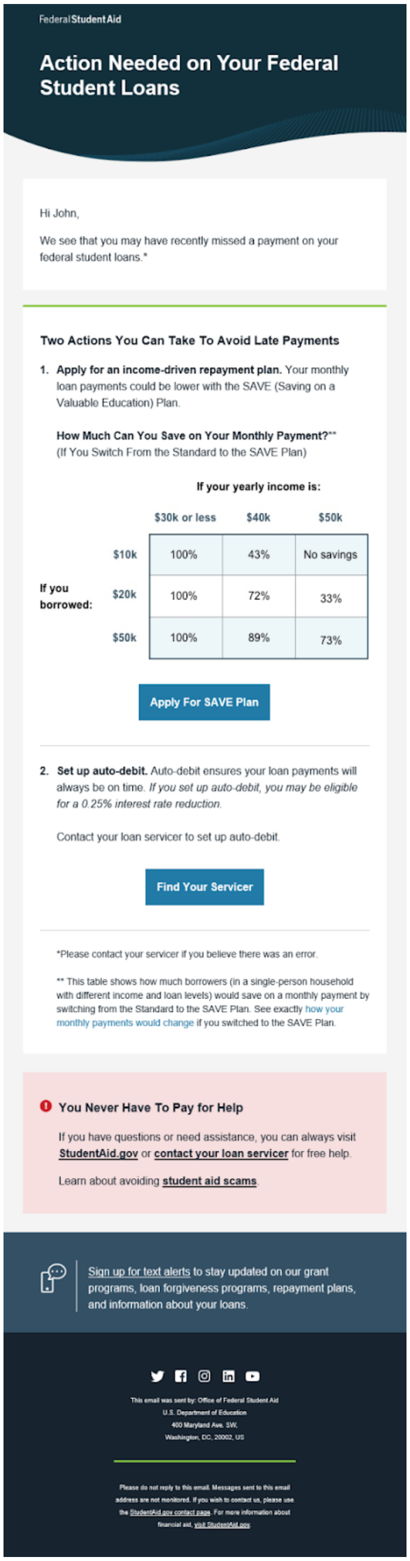

Federal Student Aid

Action Needed on Your Federal Student Loans

Hi John,

We see that you may have recently missed a payment on your federal student loans.*

Two Actions You Can Take To Avoid Late Payments

1. **Apply for an income-driven repayment plan.** Your monthly loan payments could be lower with the SAVE (Saving on a Valuable Education) Plan.

How Much Can You Save on Your Monthly Payment?**
(If You Switch From the Standard to the SAVE Plan)

		If your yearly income is:		
		$30k or less	$40k	$50k
If you borrowed:	$10k	100%	43%	No savings
	$20k	100%	72%	33%
	$50k	100%	89%	73%

Apply For SAVE Plan

2. **Set up auto-debit.** Auto-debit ensures your loan payments will always be on time. *If you set up auto-debit, you may be eligible for a 0.25% interest rate reduction.*

Contact your loan servicer to set up auto-debit.

Find Your Servicer

*Please contact your servicer if you believe there was an error.

** This table shows how much borrowers (in a single-person household with different income and loan levels) would save on a monthly payment by switching from the Standard to the SAVE Plan. See exactly how your monthly payments would change if you switched to the SAVE Plan.

You Never Have To Pay for Help

If you have questions or need assistance, you can always visit **StudentAid.gov** or **contact your loan servicer** for free help.

Learn about avoiding **student aid scams**.

Sign up for text alerts to stay updated on our grant programs, loan forgiveness programs, repayment plans, and information about your loans.

This email was sent by: Office of Federal Student Aid
U.S. Department of Education
400 Maryland Ave. SW,
Washington, DC, 20002, US

Please do not reply to this email. Messages sent to this email address are not monitored. If you wish to contact us, please use the StudentAid.gov contact page. For more information about financial aid, visit StudentAid.gov.

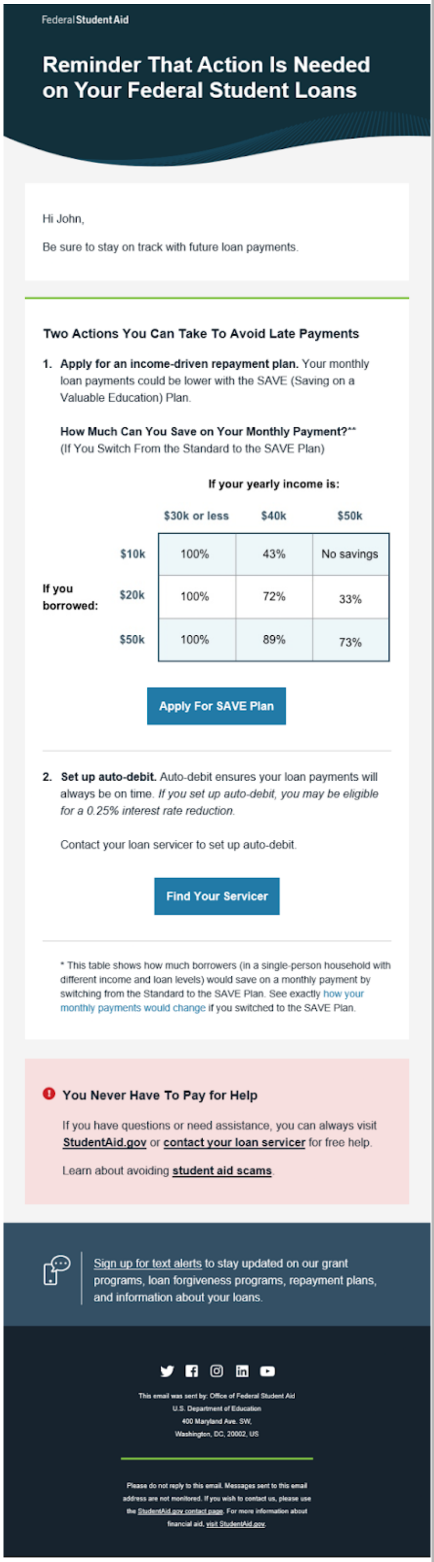

Federal Student Aid

Reminder That Action Is Needed on Your Federal Student Loans

Hi John,

Be sure to stay on track with future loan payments.

Two Actions You Can Take To Avoid Late Payments

1. **Apply for an income-driven repayment plan.** Your monthly loan payments could be lower with the SAVE (Saving on a Valuable Education) Plan.

How Much Can You Save on Your Monthly Payment?**
(If You Switch From the Standard to the SAVE Plan)

		If your yearly income is:		
		$30k or less	$40k	$50k
If you borrowed:	$10k	100%	43%	No savings
	$20k	100%	72%	33%
	$50k	100%	89%	73%

Apply For SAVE Plan

2. **Set up auto-debit.** Auto-debit ensures your loan payments will always be on time. *If you set up auto-debit, you may be eligible for a 0.25% interest rate reduction.*

Contact your loan servicer to set up auto-debit.

Find Your Servicer

* This table shows how much borrowers (in a single-person household with different income and loan levels) would save on a monthly payment by switching from the Standard to the SAVE Plan. See exactly how your monthly payments would change if you switched to the SAVE Plan.

You Never Have To Pay for Help

If you have questions or need assistance, you can always visit **StudentAid.gov** or **contact your loan servicer** for free help.

Learn about avoiding **student aid scams**.

Sign up for text alerts to stay updated on our grant programs, loan forgiveness programs, repayment plans, and information about your loans.

This email was sent by: Office of Federal Student Aid
U.S. Department of Education
400 Maryland Ave. SW,
Washington, DC, 20002, US

Please do not reply to this email. Messages sent to this email address are not monitored. If you wish to contact us, please use the StudentAid.gov contact page. For more information about financial aid, visit StudentAid.gov.

Fig. S3. Emails sent to borrowers in the "two-action, IDR % benefits email with a reminder" condition.

First Email	Second Email (sent 3 days later)

First Email

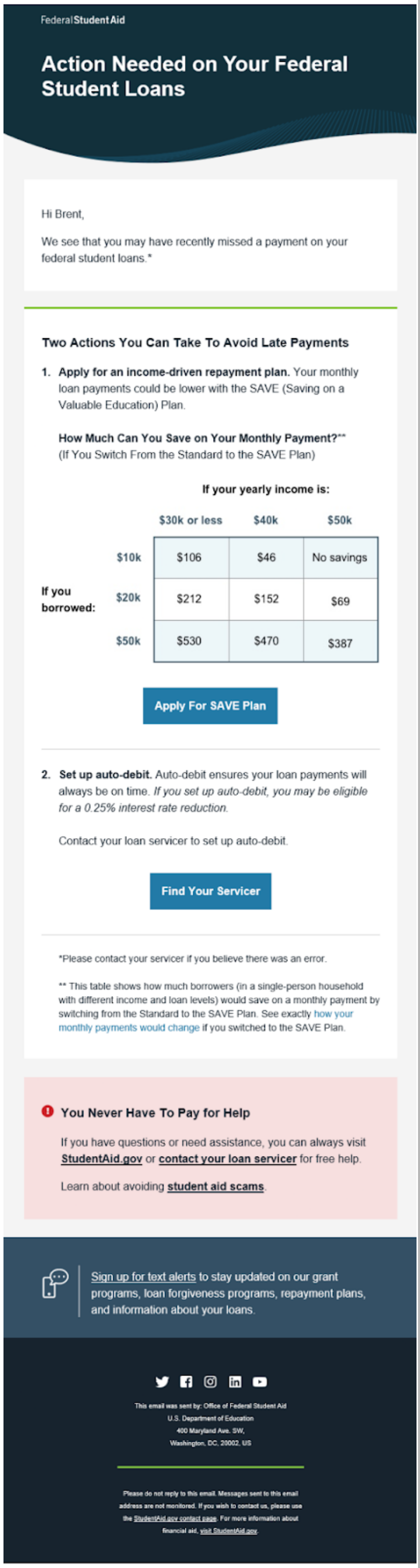

Federal Student Aid

Action Needed on Your Federal Student Loans

Hi Brent,

We see that you may have recently missed a payment on your federal student loans.*

Two Actions You Can Take To Avoid Late Payments

1. **Apply for an income-driven repayment plan.** Your monthly loan payments could be lower with the SAVE (Saving on a Valuable Education) Plan.

How Much Can You Save on Your Monthly Payment?**
(If You Switch From the Standard to the SAVE Plan)

		If your yearly income is:		
		$30k or less	$40k	$50k
If you borrowed:	$10k	$106	$46	No savings
	$20k	$212	$152	$69
	$50k	$530	$470	$387

Apply For SAVE Plan

2. **Set up auto-debit.** Auto-debit ensures your loan payments will always be on time. *If you set up auto-debit, you may be eligible for a 0.25% interest rate reduction.*

Contact your loan servicer to set up auto-debit.

Find Your Servicer

*Please contact your servicer if you believe there was an error.

** This table shows how much borrowers (in a single-person household with different income and loan levels) would save on a monthly payment by switching from the Standard to the SAVE Plan. See exactly how your monthly payments would change if you switched to the SAVE Plan.

You Never Have To Pay for Help

If you have questions or need assistance, you can always visit StudentAid.gov or contact your loan servicer for free help.

Learn about avoiding student aid scams.

Sign up for text alerts to stay updated on our grant programs, loan forgiveness programs, repayment plans, and information about your loans.

This email was sent by: Office of Federal Student Aid
U.S. Department of Education
400 Maryland Ave. SW,
Washington, DC, 20002, US

Please do not reply to this email. Messages sent to this email address are not monitored. If you wish to contact us, please use the StudentAid.gov contact page. For more information about financial aid, visit StudentAid.gov.

Second Email (sent 3 days later)

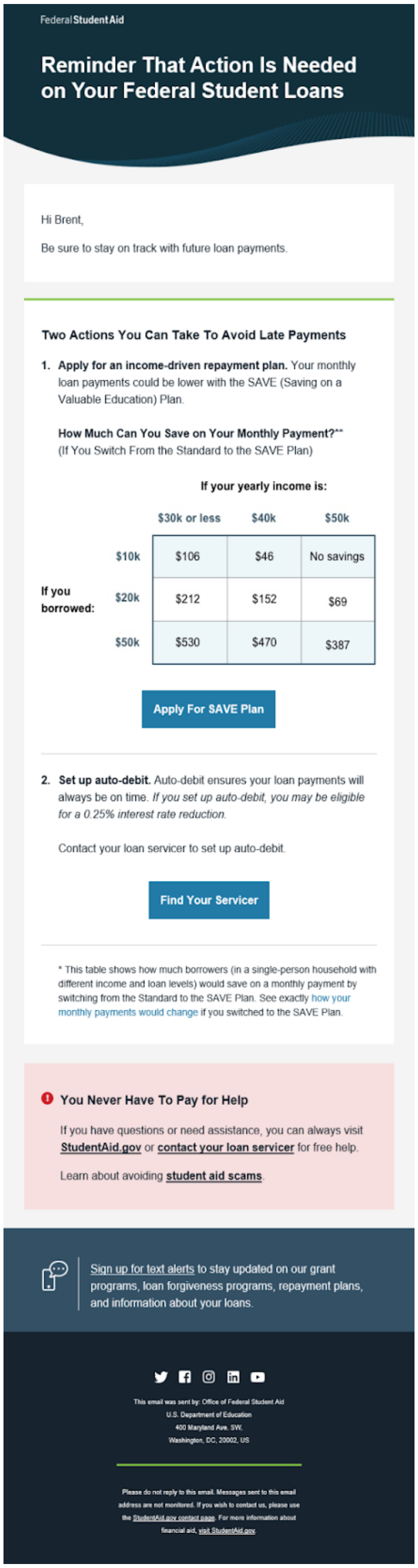

Federal Student Aid

Reminder That Action Is Needed on Your Federal Student Loans

Hi Brent,

Be sure to stay on track with future loan payments.

Two Actions You Can Take To Avoid Late Payments

1. **Apply for an income-driven repayment plan.** Your monthly loan payments could be lower with the SAVE (Saving on a Valuable Education) Plan.

How Much Can You Save on Your Monthly Payment?**
(If You Switch From the Standard to the SAVE Plan)

		If your yearly income is:		
		$30k or less	$40k	$50k
If you borrowed:	$10k	$106	$46	No savings
	$20k	$212	$152	$69
	$50k	$530	$470	$387

Apply For SAVE Plan

2. **Set up auto-debit.** Auto-debit ensures your loan payments will always be on time. *If you set up auto-debit, you may be eligible for a 0.25% interest rate reduction.*

Contact your loan servicer to set up auto-debit.

Find Your Servicer

* This table shows how much borrowers (in a single-person household with different income and loan levels) would save on a monthly payment by switching from the Standard to the SAVE Plan. See exactly how your monthly payments would change if you switched to the SAVE Plan.

You Never Have To Pay for Help

If you have questions or need assistance, you can always visit StudentAid.gov or contact your loan servicer for free help.

Learn about avoiding student aid scams.

Sign up for text alerts to stay updated on our grant programs, loan forgiveness programs, repayment plans, and information about your loans.

This email was sent by: Office of Federal Student Aid
U.S. Department of Education
400 Maryland Ave. SW,
Washington, DC, 20002, US

Please do not reply to this email. Messages sent to this email address are not monitored. If you wish to contact us, please use the StudentAid.gov contact page. For more information about financial aid, visit StudentAid.gov.

Fig. S4. Emails sent to borrowers in the "two-action, IDR $ benefits email with a reminder" condition.

First Email

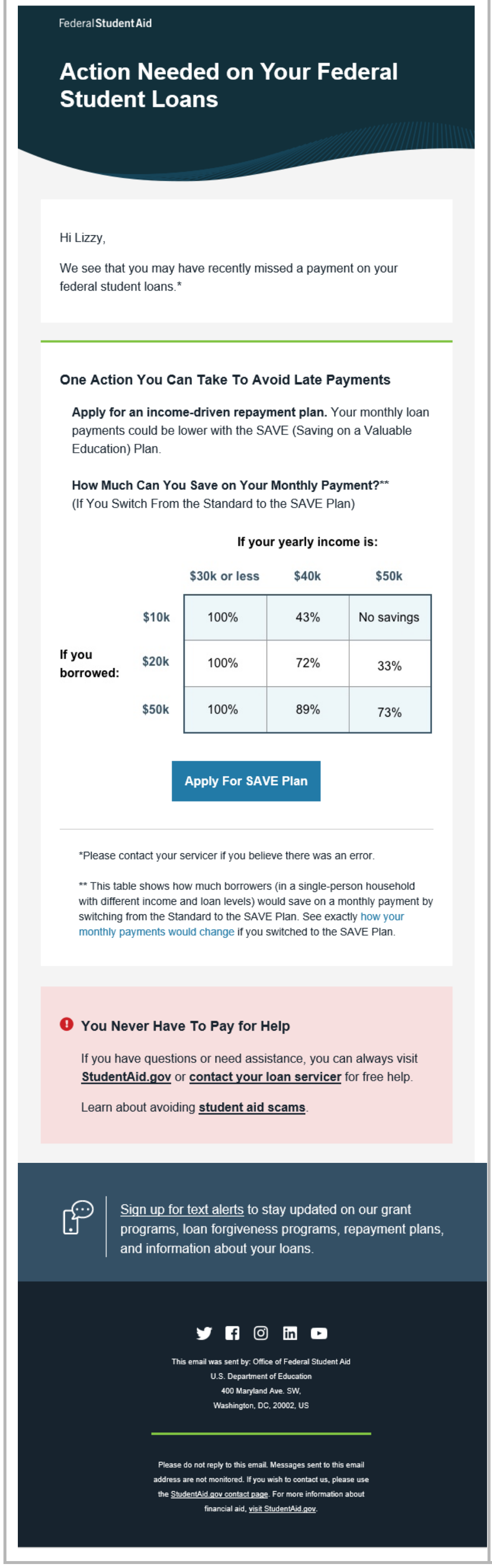

Federal Student Aid

Action Needed on Your Federal Student Loans

Hi Lizzy,

We see that you may have recently missed a payment on your federal student loans.*

One Action You Can Take To Avoid Late Payments

Apply for an income-driven repayment plan. Your monthly loan payments could be lower with the SAVE (Saving on a Valuable Education) Plan.

How Much Can You Save on Your Monthly Payment?**
(If You Switch From the Standard to the SAVE Plan)

If you borrowed:	If your yearly income is: $30k or less	$40k	$50k
$10k	100%	43%	No savings
$20k	100%	72%	33%
$50k	100%	89%	73%

Apply For SAVE Plan

*Please contact your servicer if you believe there was an error.

** This table shows how much borrowers (in a single-person household with different income and loan levels) would save on a monthly payment by switching from the Standard to the SAVE Plan. See exactly how your monthly payments would change if you switched to the SAVE Plan.

You Never Have To Pay for Help

If you have questions or need assistance, you can always visit **StudentAid.gov** or **contact your loan servicer** for free help.

Learn about avoiding **student aid scams**.

Sign up for text alerts to stay updated on our grant programs, loan forgiveness programs, repayment plans, and information about your loans.

This email was sent by: Office of Federal Student Aid
U.S. Department of Education
400 Maryland Ave. SW,
Washington, DC, 20002, US

Please do not reply to this email. Messages sent to this email address are not monitored. If you wish to contact us, please use the StudentAid.gov contact page. For more information about financial aid, visit StudentAid.gov.

Second Email (sent 3 days later)

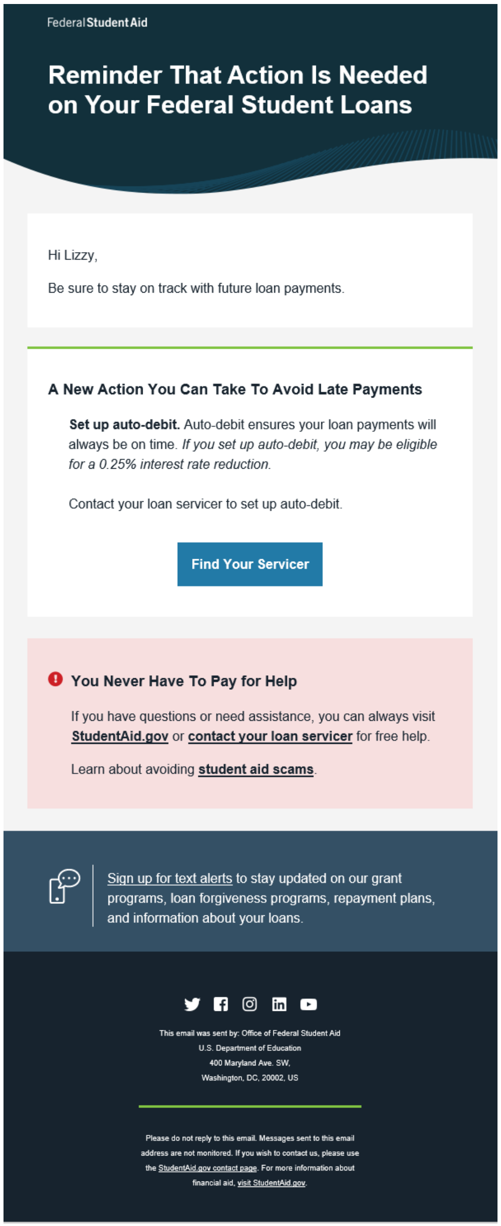

Federal Student Aid

Reminder That Action Is Needed on Your Federal Student Loans

Hi Lizzy,

Be sure to stay on track with future loan payments.

A New Action You Can Take To Avoid Late Payments

Set up auto-debit. Auto-debit ensures your loan payments will always be on time. *If you set up auto-debit, you may be eligible for a 0.25% interest rate reduction.*

Contact your loan servicer to set up auto-debit.

Find Your Servicer

You Never Have To Pay for Help

If you have questions or need assistance, you can always visit **StudentAid.gov** or **contact your loan servicer** for free help.

Learn about avoiding **student aid scams**.

Sign up for text alerts to stay updated on our grant programs, loan forgiveness programs, repayment plans, and information about your loans.

This email was sent by: Office of Federal Student Aid
U.S. Department of Education
400 Maryland Ave. SW,
Washington, DC, 20002, US

Please do not reply to this email. Messages sent to this email address are not monitored. If you wish to contact us, please use the StudentAid.gov contact page. For more information about financial aid, visit StudentAid.gov.

Fig. S5. Emails sent to borrowers in the “one-action, IDR % benefits email with a reminder” condition.

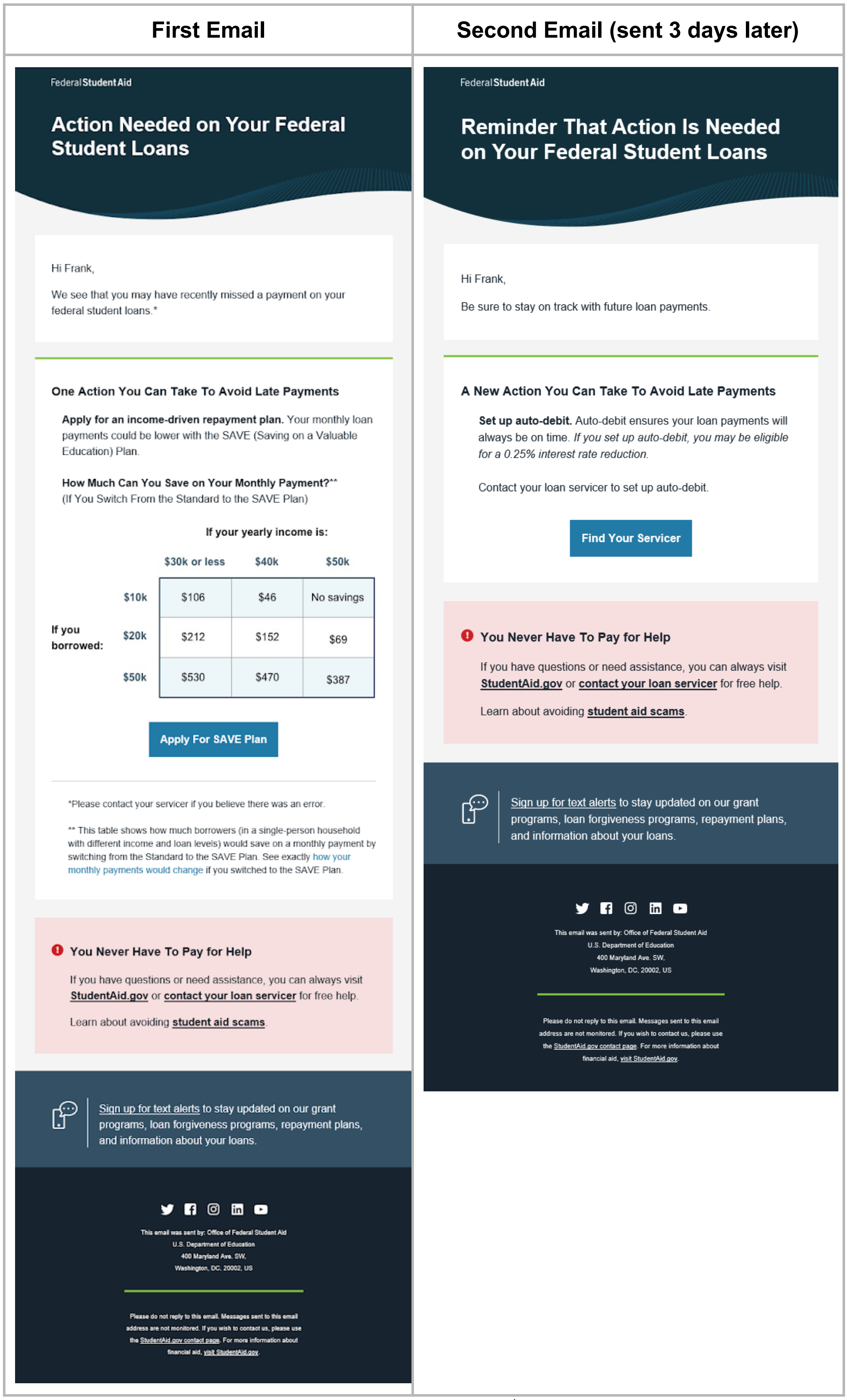

First Email	Second Email (sent 3 days later)

First Email

FederalStudentAid

Action Needed on Your Federal Student Loans

Hi Frank,

We see that you may have recently missed a payment on your federal student loans.*

One Action You Can Take To Avoid Late Payments

Apply for an income-driven repayment plan. Your monthly loan payments could be lower with the SAVE (Saving on a Valuable Education) Plan.

How Much Can You Save on Your Monthly Payment?**
(If You Switch From the Standard to the SAVE Plan)

		If your yearly income is: $30k or less	$40k	$50k
If you borrowed:	$10k	$106	$46	No savings
	$20k	$212	$152	$69
	$50k	$530	$470	$387

Apply For SAVE Plan

*Please contact your servicer if you believe there was an error.

** This table shows how much borrowers (in a single-person household with different income and loan levels) would save on a monthly payment by switching from the Standard to the SAVE Plan. See exactly how your monthly payments would change if you switched to the SAVE Plan.

You Never Have To Pay for Help

If you have questions or need assistance, you can always visit **StudentAid.gov** or **contact your loan servicer** for free help.

Learn about avoiding **student aid scams**.

Sign up for text alerts to stay updated on our grant programs, loan forgiveness programs, repayment plans, and information about your loans.

This email was sent by: Office of Federal Student Aid
U.S. Department of Education
400 Maryland Ave. SW,
Washington, DC, 20002, US

Please do not reply to this email. Messages sent to this email address are not monitored. If you wish to contact us, please use the StudentAid.gov contact page. For more information about financial aid, visit StudentAid.gov.

Second Email (sent 3 days later)

FederalStudentAid

Reminder That Action Is Needed on Your Federal Student Loans

Hi Frank,

Be sure to stay on track with future loan payments.

A New Action You Can Take To Avoid Late Payments

Set up auto-debit. Auto-debit ensures your loan payments will always be on time. *If you set up auto-debit, you may be eligible for a 0.25% interest rate reduction.*

Contact your loan servicer to set up auto-debit.

Find Your Servicer

You Never Have To Pay for Help

If you have questions or need assistance, you can always visit **StudentAid.gov** or **contact your loan servicer** for free help.

Learn about avoiding **student aid scams**.

Sign up for text alerts to stay updated on our grant programs, loan forgiveness programs, repayment plans, and information about your loans.

This email was sent by: Office of Federal Student Aid
U.S. Department of Education
400 Maryland Ave. SW,
Washington, DC, 20002, US

Please do not reply to this email. Messages sent to this email address are not monitored. If you wish to contact us, please use the StudentAid.gov contact page. For more information about financial aid, visit StudentAid.gov.

Fig. S6. Emails sent to borrowers in the "one-action, IDR $ benefits email with a reminder" condition.

Tables

Table S1. Summary statistics and balance tests of key variables by condition assignment.

	Observations		All Conditions		Control		Two-Action, % Benefits		Two-Action, % Benefits, w/ Reminder		Two-Action, $ Benefits, w/ Reminder		One-Action, % Benefits, w/ Reminder		One-Action, $ Benefits, w/ Reminder		F-Test of Equality on Borrower Covariate Values		F-Test of Equality on Proportion of Missing Values	
	n (Millions)	% Missing	Mean	SD	Mean	SD	Mean	SD	Mean	SD	Mean	SD	Mean	SD	Mean	SD	F-stat	p-value	F-stat	p-value
**Borrower Demographics**																				
Age	12.8	0.01	37.8	12.0	37.8	12.0	37.8	12.0	37.8	12.0	37.9	12.0	37.8	12.0	37.8	12.0	0.84	0.518	2.72	0.018
% Male	11.4	10.41	37.9		37.8		38.0		38.0		37.9		37.9		37.9		0.97	0.431	0.94	0.455
% Who Reached 4 Yrs of Undergraduate College[1]	12.7	0.38	39.4		39.4		39.4		39.4		39.5		39.4		39.4		0.61	0.692	0.36	0.875
% Who Graduated from Last School Attended	12.8	0.01	36.0		35.9		36.0		36.0		36.0		36.0		36.0		0.70	0.623	2.62	0.022
**Borrower Loan Features**																				
Days Since Last Federal Student Loan	12.8	0.03	2,354	1,597	2,355	1,591	2,355	1,598	2,354	1,596	2,355	1,597	2,354	1,597	2,354	1,597	0.39	0.859	0.47	0.796
Total Funds Obtained from Federal Student Loans ($)	12.8	0.03	32,801	42,588	32,678	42,320	32,802	42,609	32,773	42,557	32,799	42,571	32,816	42,657	32,827	42,572	0.90	0.479	0.85	0.516
Current Outstanding Loan Balance ($)	12.8	0.02	34,655	49,782	34,517	49,347	34,661	49,801	34,628	49,729	34,651	49,822	34,661	49,801	34,686	49,802	0.75	0.584	1.32	0.252
Monthly Payment Owed ($)	12.8	0.02	340	528	340	519	340	529	340	517	340	518	341	534	340	540	0.85	0.517	0.65	0.658
% Who Use Largest Loan Servicer	12.6	1.24	39.5		39.7		39.5		39.5		39.5		39.5		39.5		0.80	0.550	1.13	0.343
% Who Use 2nd Largest Loan Servicer	12.6	1.24	26.8		26.8		26.8		26.8		26.8		26.8		26.8		0.16	0.976	1.13	0.343
% Who Use 3nd Largest Loan Servicer	12.6	1.24	17.0		16.9		17.0		17.0		17.0		17.0		17.0		0.95	0.448	1.13	0.343
% Who Use 4th Largest Loan Servicer	12.6	1.24	15.9		15.9		15.9		15.9		15.9		16.0		15.9		0.90	0.478	1.13	0.343
% on a Standard Repayment Plan (as of 3/2020)	8.8	31.44	48.7		48.8		48.7		48.7		48.7		48.7		48.7		0.60	0.703	1.39	0.225
% With 1+ Parent Plus Loans	12.8	0.00	12.6		12.6		12.6		12.6		12.6		12.6		12.6		0.79	0.556	n/a[3]	n/a[3]
% With 1+ Consolidation Loans	12.8	0.02	19.2		19.3		19.2		19.3		19.3		19.2		19.2		0.52	0.762	1.32	0.252
**Borrower's Actions**																				
% Who Ever Had a Late Loan Payment ≥ 60 Days	8.6	32.38	63.1		63.2		63.1		63.1		63.1		63.0		63.1		1.18	0.317	1.33	0.250
% Who Ever Had a Loan Default	12.8	0.01	21.1		21.1		21.1		21.1		21.1		21.1		21.1		0.35	0.883	2.62	0.022
% Who Were Enrolled in IDR (as of 2/2020)	11.3	11.57	17.2		17.3		17.2		17.3		17.2		17.2		17.2		0.60	0.697	0.50	0.777
% Who Were Enrolled in Auto-debit (as of 2/2020)	11.3	11.57	9.2		9.3		9.2		9.2		9.2		9.2		9.3		1.42	0.212	0.50	0.777
% of Months With a Late Loan Payment ≥ 30 Days[2]	8.0	37.27	42.8	34.9	42.8	34.9	42.8	34.9	42.8	34.9	42.7	34.9	42.7	34.9	42.8	34.9	1.23	0.292	1.59	0.159

Notes: Means and standard deviations are reported for continuous covariates, while only means are reported for binary variables. All variables are as of August 2023 unless otherwise noted. To check that random assignment was successful, we conducted balance checks on 20 borrower covariates, shown above. To test for differences in borrower covariate values across conditions (see column “F-Test of Equality on Borrower Covariate Values”), we regressed each of the 20 borrower covariates on indicators of assignment for each of our five treatment conditions (with the indicator for the control condition omitted) using an OLS regression with HC1 robust standard errors. Then, for each borrower covariate, we conducted an F-test to test the null hypothesis that the coefficients for all treatment conditions were equal to zero. To test for differential rates of missing values across covariates (see column “F-Test of Equality on Proportion of Missing Values”), we ran the same procedure for the balance checks on borrower covariate values, but we replaced the 20 borrower covariates with 20 indicators for whether each of the 20 borrower covariates took on a missing value (e.g., the borrower’s age covariate was replaced with an indicator for whether the borrower’s age was missing).

Footnotes:
1. Information on maximum grade level is based on the borrower’s last reported FAFSA
2. The % of months for ever having a loan payment past due for 30 days or more (30-day delinquency) from January 2015 to March 2020 (prior to the repayment pause)
3. Our control variable for whether a borrower had at least one Parent PLUS loan did not have missing value

Table S2. Regression-estimated impact of recommending two recommended actions repeatedly (vs. one of two recommended action sequentially) on whether a borrower clicked on a link to apply for an IDR plan (Model 1), clicked on a link to sign up for auto debit or find their servicer (Model 2), and clicked on a link either to apply for an IDR plan, sign up for auto debit, or find their servicer (Model 3).

	**Model 1**	**Model 2**	**Model 3**
Two-Action Email	0.01435***	-0.01304***	0.00213***
	(0.00014)	(0.00013)	(0.00018)
Are Controls Included?	Yes	Yes	Yes
Number of Observations	9,865,200	9,865,200	9,865,200
Adjusted R-Squared	0.01	0.01	0.01

*** $p < 0.001$, ** $p < 0.01$, * $p < 0.05$, $^{+}$ $p < 0.10$

Notes: This table reports the results of three ordinary least squares (OLS) regressions including the subset of borrowers assigned to one of four conditions in our experiment: the “Two-Action, IDR % Benefits Email with Reminder” condition, the “Two-Action, IDR $ Benefits Email with Reminder” condition, the “One-Action, IDR % Benefits Email with Reminder” condition and the “One-Action, IDR $ Benefits Email with Reminder” condition. The dependent variables are a binary indicator for whether a borrower clicked on a link to apply for an IDR plan in the first set of intervention emails received (Model 1), a binary indicator for whether a borrower clicked on a link to sign up for auto debit or “contact [their] servicer” in the first set of intervention emails received (Model 2), and a binary indicator for whether a borrower clicked on a link to apply for an IDR plan, sign up for auto debit, or “contact [their] servicer” in the first set of intervention emails received (Model 3). The primary predictor variables are indicators for whether a borrower received communications that described IDR benefits in % (vs. $) terms and whether a borrower received communications that recommended two actions repeatedly (vs. communications that recommended one of two recommended actions sequentially). For all models, control variables were included for borrower age as of Aug 2023, an indicator for whether the borrower was male, indicators for education grade level reported on the borrower’s last FAFSA application as of Aug 2023, an indicator for whether the borrower graduated from their last reported educational institution attended as of Aug 2023, indicators for the borrower’s state of residence as of Aug 2023, the number of days that elapsed between when a borrower took out their last loan as of Aug 2023 and when they first received an intervention email, the total funds loaned to the borrower as of Aug 2023, the borrower’s current outstanding loan balance as of Aug 2023, the borrower’s monthly loan payment amount owed as of Aug 2023, an indicator for a borrower’s loan servicer as of Aug 2023, an indicator for the borrower’s last loan repayment type prior to the repayment pause as of March 2020 (e.g., standard repayment plan, graduated repayment plan, etc.), indicators for each loan type owned by borrower as of Aug 2023 (i.e., Parent PLUS loans, Consolidation loans), an indicator for ever having a loan payment past due for 60 days or more (60-day delinquency) as of Aug 2023, an indicator for ever having a loan in default as of Aug 2023, an indicator for whether the borrower was enrolled in an IDR (income-driven repayment) plan in Feb 2020 (prior to the repayment pause), an indicator for whether the borrower was signed up for auto debit in Feb 2020 (prior to the repayment pause), the % of months the borrower ever had a loan payment past due for 30 days or more (30-day delinquency) from January 2015 to March 2020 (prior to the repayment pause), and indicators for whether the participants first missed a payment on all possible missed payment dates (prior to eligibility for study participation being determined and prior to receiving an intervention treatment email). If a control variable contained missing data, we addressed it in two steps: i) for binary or categorical variables, missing values were replaced with zero; for continuous variables, missing values were replaced with the mean that variable took on in non-missing observations; ii) we added an indicator variable to the regression, coded as one if the original variable was missing and zero otherwise. All standard errors shown are HC1 robust standard errors. All sample sizes reported above are rounded to the nearest hundred borrowers in accordance with disclosure policies required by the Department of Education.

Table S3. Significant heterogeneous treatment effects for borrower covariates.

Comparison	DV	Effect	P-value on Interaction Coefficient
Behaviorally-Informed Emails (vs. Control Email)	Increase in IDR Applications (within 90 days)	Larger effect for female borrowers	0.000
		Larger effect for borrowers with less days elapsed since last federal student loan	0.001
		Larger effect for borrowers who were not on the standard repayment plan	0.002
		Larger effect for borrowers who were not enrolled in auto-debit (as of 2/2020)	0.007
		Larger effect for borrowers who were enrolled in IDR (as of 2/2020)	0.018
		Larger effect for borrowers who have a higher current outstanding loan balance	0.047
	Increase in At Least One Payment (within 90 days)	Larger effect for borrowers who do not have a Parent PLUS loan	0.014
		Larger effect for borrowers who have the 2nd largest loan servicer	0.016
	Reduction in 60-Day Delinquency (within 180 days)	Larger effect for borrowers who were enrolled in IDR (as of 2/2020)	0.000
		Larger effect for borrowers who ever had a late loan payment ≥ 270 days	0.017
		Larger effect for borrowers who do not have the 4th largest loan servicer	0.029
Reminder Email (vs. No Reminder Email)	Increase in IDR Applications (within 90 days)	Larger effect for female borrowers	0.000
		Larger effect for borrowers who were enrolled in IDR (as of 2/2020)	0.000
		Larger effect for borrowers who have a higher current outstanding loan balance	0.000
		Larger effect for borrowers with less days elapsed since last federal student loan	0.000
		Larger effect for borrowers with higher monthly payments owed	0.001
		Larger effect for borrowers who obtained more total funds from federal student loans	0.001
		Larger effect for older borrowers	0.001
		Larger effect for borrowers who have a consolidation loan	0.003
		Larger effect for borrowers who were not on the standard repayment plan	0.004
		Larger effect for borrowers who were not enrolled in auto-debit (as of 2/2020)	0.008
	Increase in Auto-debit Signups (within 90 days)	Larger effect for borrowers who never had a late loan payment ≥ 60 days	0.003
		Larger effect for borrowers who do not have the 3rd largest loan servicer	0.011
		Larger effect for borrowers who have the 4th largest loan servicer	0.023
		Larger effect for borrowers with a lower % of months with a late loan payment ≥ 30 days[1]	0.036
	Increase in At Least One Payment (within 90 days)	Larger effect for borrowers who never had a late loan payment ≥ 60 days	0.039
	Reduction in 60-Day Delinquency (within 180 days)	Larger effect for borrowers who reached 4 years of undergraduate college[2]	0.032
		Larger effect for borrowers who obtained more total funds from federal student loans	0.037
IDR % Benefits (vs. IDR $ Benefits)	Increase in IDR Applications (within 90 days)	Larger effect for female borrowers	0.000
		Larger effect for borrowers who have a higher current outstanding loan balance	0.001
		Larger effect for borrowers with less days elapsed since last federal student loan	0.001
		Larger effect for borrowers who obtained more total funds from federal student loans	0.009
		Larger effect for borrowers who were not enrolled in auto-debit (as of 2/2020)	0.010
		Larger effect for borrowers who were enrolled in IDR (as of 2/2020)	0.020
		Larger effect for borrowers who did not graduate from their last school attended	0.027
	Increase in Auto-debit Signups (within 90 days)	Larger effect for borrowers who have the 4th largest loan servicer	0.002
		Larger effect for borrowers who do not have a Parent PLUS loan	0.036
	At Least One Payment (within 90 days)	Larger effect for borrowers who do not have a Parent PLUS loan	0.016
		Larger effect for borrowers who were not on the standard repayment plan	0.039
Two-action Emails (vs. One-action Emails)	Increase in IDR Applications (within 90 days)	Larger effect for borrowers who were enrolled in IDR (as of 2/2020)	0.000
		Larger effect for female borrowers	0.000
		Larger effect for borrowers who were not on the standard repayment plan	0.000
		Larger effect for borrowers who have the largest loan servicer	0.000
		Larger effect for borrowers with less days elapsed since last federal student loan	0.002
		Larger effect for borrowers with higher monthly payments owed	0.008
		Larger effect for borrowers who have a higher current outstanding loan balance	0.012
		Larger effect for borrowers who do not have the 3rd largest loan servicer	0.031
		Larger effect for borrowers who obtained more total funds from federal student loans	0.034
		Larger effect for older borrowers	0.042
		Larger effect for borrowers who have a consolidation loan	0.047
	Increase in Auto-debit Signups (within 90 days)	Larger effect for borrowers who have the 2nd largest loan servicer	0.006
		Larger effect for borrowers with lower monthly payments owed	0.048
	Increase in At Least One Payment (within 90 days)	Larger effect for borrowers with lower monthly payments owed	0.016
	Reduction in 60-Day Delinquency (within 180 days)	Larger effect for borrowers who did not graduate from their last school attended	0.025
		Larger effect for borrowers with lower monthly payments owed	0.039

Notes: This table presents the results of statistically significant interactions at the 0.05 alpha significance level for all heterogeneous treatment effects detected across our four interventions and four dependent variables without correcting for multiple comparisons. We explored the possible heterogeneity for all 20 borrower covariates listed in Table 1. To probe for heterogeneity in our effects as a function of a given borrower attribute (e.g., borrower age), we added an interaction (or set of interactions) between the primary treatment indicator(s) in each regression (in Tables 2-4 in the main text) and a given borrower attribute of interest (e.g., age). To handle missing data, we also added an interaction (or set of interactions) between each treatment indicator in the regression and an indicator for a missing value for the borrower attribute of interest (e.g., age missing).

Footnotes:
1. The % of months for ever having a loan payment past due for 30 days or more (30-day delinquency) from January 2015 to March 2020 (prior to the repayment pause)
2. Information on maximum grade level is based on the borrower’s last reported FAFSA

Table S4. Logistic regression-estimated impact of the pooled set of behaviorally-informed emails on whether a borrower applied for an IDR plan within 90 days of receiving an intervention email (Model 1), signed up for auto debit within 90 days of receiving an intervention email (Model 2), made at least one loan payment within 90 days of receiving an intervention email (Model 3), and entered into 60-day delinquency within 180 days of receiving an intervention email (Model 4).

	**Model 1**	**Model 2**	**Model 3**	**Model 4**
Behaviorally-Informed Emails	0.17121***	0.01970+	0.01725***	-0.02390***
	(0.00892)	(0.01089)	(0.00483)	(0.00472)
Are Controls Included?	Yes	Yes	Yes	Yes
Number of Observations	10,768,400	12,495,300	12,766,300	12,766,300
Log Likelihood	-2,774,641	-1,774,411	-6,653,545	-6,917,346
BIC	5,552,099	3,551,666	13,310,067	13,837,687

*** $p < 0.001$, ** $p < 0.01$, * $p < 0.05$, + $p < 0.10$

Notes: This table reports the results of four logistic regressions including all borrowers in our experiment. The dependent variables are a binary indicator for whether a borrower applied for an IDR plan within 90 days of first receiving an intervention email (Model 1), a binary indicator for whether a borrower signed up for auto debit within 90 days of first receiving an intervention email (Model 2), a binary indicator for whether a borrower made at least one loan payment within 90 days of first receiving an intervention email (Model 3, and a binary indicator for whether a borrower lapsed into 60-day delinquency within 180 days of first receiving an intervention email. The primary predictor variable is an indicator for assignment to a condition in which a borrower received a behaviorally-informed email (with the control condition as the comparison group). For all models, control variables were included for borrower age as of Aug 2023, an indicator for whether the borrower was male, indicators for education grade level reported on the borrower's last FAFSA application as of Aug 2023, an indicator for whether the borrower graduated from their last reported educational institution attended as of Aug 2023, indicators for the borrower's state of residence as of Aug 2023, the number of days that elapsed between when a borrower took out their last loan as of Aug 2023 and when they first received an intervention email, the total funds loaned to the borrower as of Aug 2023, the borrower's current outstanding loan balance as of Aug 2023, the borrower's monthly loan payment amount owed as of Aug 2023, an indicator for a borrower's loan servicer as of Aug 2023, an indicator for the borrower's last loan repayment type prior to the repayment pause as of March 2020 (e.g., standard repayment plan, graduated repayment plan, etc.), indicators for each loan type owned by borrower as of Aug 2023 (i.e., Parent PLUS loans, Consolidation loans), an indicator for ever having a loan payment past due for 60 days or more (60-day delinquency) as of Aug 2023, an indicator for ever having a loan in default as of Aug 2023, an indicator for whether the borrower was enrolled in an IDR (income-driven repayment) plan in Feb 2020 (prior to the repayment pause), an indicator for whether the borrower was signed up for auto debit in Feb 2020 (prior to the repayment pause), the % of months the borrower ever had a loan payment past due for 30 days or more (30-day delinquency) from January 2015 to March 2020 (prior to the repayment pause), and indicators for whether the participants first missed a payment on all possible missed payment dates (prior to eligibility for study participation being determined and prior to receiving an intervention treatment email). If a control variable contained missing data, we addressed it in two steps: i) for binary or categorical variables, missing values were replaced with zero; for continuous variables, missing values were replaced with the mean that variable took on in non-missing observations; ii) we added an indicator variable to the regression, coded as one if the original variable was missing and zero otherwise. For Model 1, borrowers who had already signed up for a Saving on a Valuable Education (SAVE) plan as of the send date of our first intervention email message and borrowers who were ineligible to apply for a SAVE plan were excluded from our analysis. For Model 2, borrowers who had signed up for auto debit on all their loans as of the send date of our first intervention email message were excluded from our analysis. All sample sizes reported above are rounded to the nearest hundred borrowers in accordance with disclosure policies required by the Department of Education.

Table S5. Logistic regression-estimated impact of sending a follow-up email three days after a missed payment email on a borrower applying to an IDR plan within 90 days of receiving intervention (Model 1), signing up for auto debit within 90 days of receiving intervention (Model 2), making at least one payment within 90 days of receiving intervention (Model 3), and entering into 60-day delinquency within 180 days of receiving intervention (Model 4).

	**Model 1**	**Model 2**	**Model 3**	**Model 4**
Reminder Email	0.09541***	0.02155***	0.02802***	-0.03198***
	(0.00370)	(0.00486)	(0.00215)	(0.00210)
Are Controls Included?	Yes	Yes	Yes	Yes
Number of Observations	4,220,200	4,897,600	5,003,700	5,003,700
Log Likelihood	-1,102,617	-690,550	-2,605,348	-2,709,246
BIC	2,207,872	1,383,765	5,213,427	5,421,222

*** $p < 0.001$, ** $p < 0.01$, * $p < 0.05$, $^{+}$ $p < 0.10$

Notes: This table reports the results of four logistic regressions including the subset of borrowers assigned to one of two conditions in our experiment: the “Two-Action, IDR % Benefits Email with Reminder” condition or the “Two-Action, IDR % Benefits Email” condition. The dependent variables are a binary indicator for whether a borrower applied for an IDR plan within 90 days of first receiving an intervention email (Model 1), a binary indicator for whether a borrower signed up for auto debit within 90 days of first receiving an intervention email (Model 2), a binary indicator for whether a borrower made at least one loan payment within 90 days of first receiving an intervention email (Model 3, and a binary indicator for whether a borrower lapsed into 60-day delinquency within 180 days of first receiving an intervention email. The primary predictor variable is an indicator for assignment to the “Two-Action, % IDR Benefits Email with Reminder” condition. For all models, control variables were included for borrower age as of Aug 2023, an indicator for whether the borrower was male, indicators for education grade level reported on the borrower’s last FAFSA application as of Aug 2023, an indicator for whether the borrower graduated from their last reported educational institution attended as of Aug 2023, indicators for the borrower’s state of residence as of Aug 2023, the number of days that elapsed between when a borrower took out their last loan as of Aug 2023 and when they first received an intervention email, the total funds loaned to the borrower as of Aug 2023, the borrower’s current outstanding loan balance as of Aug 2023, the borrower’s monthly loan payment amount owed as of Aug 2023, an indicator for a borrower’s loan servicer as of Aug 2023, an indicator for the borrower’s last loan repayment type prior to the repayment pause as of March 2020 (e.g., standard repayment plan, graduated repayment plan, etc.), indicators for each loan type owned by borrower as of Aug 2023 (i.e., Parent PLUS loans, Consolidation loans), an indicator for ever having a loan payment past due for 60 days or more (60-day delinquency) as of Aug 2023, an indicator for ever having a loan in default as of Aug 2023, an indicator for whether the borrower was enrolled in an IDR (income-driven repayment) plan in Feb 2020 (prior to the repayment pause), an indicator for whether the borrower was signed up for auto debit in Feb 2020 (prior to the repayment pause), the % of months the borrower ever had a loan payment past due for 30 days or more (30-day delinquency) from January 2015 to March 2020 (prior to the repayment pause), and indicators for whether the participants first missed a payment on all possible missed payment dates (prior to eligibility for study participation being determined and prior to receiving an intervention treatment email). If a control variable contained missing data, we addressed it in two steps: i) for binary or categorical variables, missing values were replaced with zero; for continuous variables, missing values were replaced with the mean that variable took on in non-missing observations; ii) we added an indicator variable to the regression, coded as one if the original variable was missing and zero otherwise. For Model 1, borrowers who had already signed up for a Saving on a Valuable Education (SAVE) plan as of the send date of our first intervention email message and borrowers who were ineligible to apply for a SAVE plan were excluded from our analysis. For Model 2, borrowers who had signed up for auto debit on all their loans as of the send date of our first intervention email message were excluded from our analysis. All sample sizes reported above are rounded to the nearest hundred borrowers in accordance with disclosure policies required by the Department of Education.

Table S6. Logistic regression-estimated impact of describing IDR benefits in % (vs. $) terms and recommending two recommended actions repeatedly (vs. one of two recommended action sequentially) on a borrower applying to an IDR plan within 90 days of receiving intervention (Model 1), signing up for auto debit within 90 days of receiving intervention (Model 2), making at least one payment within 90 days of receiving intervention (Model 3), and entering into 60-day delinquency within 180 days of receiving intervention (Model 4).

	**Model 1**	**Model 2**	**Model 3**	**Model 4**
IDR Benefits Described in %	0.04930***	-0.00358	0.00663***	-0.00787***
	(0.00262)	(0.00341)	(0.00152)	(0.00148)
Two-Action Email	0.05787***	-0.00251	-0.00103	-0.00290⁺
	(0.00262)	(0.00341)	(0.00152)	(0.00148)
Are Controls Included?	Yes	Yes	Yes	Yes
Number of Observations	8,442,700	9,796,100	10,008,800	10,008,800
Log Likelihood	-2,190,606	-1,396,957	-5,224,193	-5,431,783
BIC	4,384,004	2,796,730	10,451,303	10,866,531

*** $p < 0.001$, ** $p < 0.01$, * $p < 0.05$, ⁺ $p < 0.10$

Notes: This table reports the results of four logistic regressions including the subset of borrowers assigned to one of four conditions in our experiment: the “Two-Action, % IDR Benefits Email with Reminder” condition, the “Two-Action, $ IDR Benefits Email with Reminder” condition, the “One-Action, % IDR Benefits Email with Reminder” condition and the “One-Action, $ IDR Benefits Email with Reminder” condition. The dependent variables are a binary indicator for whether a borrower applied for an IDR plan within 90 days of first receiving an intervention email (Model 1), a binary indicator for whether a borrower signed up for auto debit within 90 days of first receiving an intervention email (Model 2), a binary indicator for whether a borrower made at least one loan payment within 90 days of first receiving an intervention email (Model 3, and a binary indicator for whether a borrower lapsed into 60-day delinquency within 180 days of first receiving an intervention email. The primary predictor variables are indicators for whether a borrower received communications that described IDR benefits in % (vs. $) terms and whether a borrower received communications that recommended two actions repeatedly (vs. communications that recommended one of two recommended actions sequentially). For all models, control variables were included for borrower age as of Aug 2023, an indicator for whether the borrower was male, indicators for education grade level reported on the borrower’s last FAFSA application as of Aug 2023, an indicator for whether the borrower graduated from their last reported educational institution attended as of Aug 2023, indicators for the borrower’s state of residence as of Aug 2023, the number of days that elapsed between when a borrower took out their last loan as of Aug 2023 and when they first received an intervention email, the total funds loaned to the borrower as of Aug 2023, the borrower’s current outstanding loan balance as of Aug 2023, the borrower’s monthly loan payment amount owed as of Aug 2023, an indicator for a borrower’s loan servicer as of Aug 2023, an indicator for the borrower’s last loan repayment type prior to the repayment pause as of March 2020 (e.g., standard repayment plan, graduated repayment plan, etc.), indicators for each loan type owned by borrower as of Aug 2023 (i.e., Parent PLUS loans, Consolidation loans), an indicator for ever having a loan payment past due for 60 days or more (60-day delinquency) as of Aug 2023, an indicator for ever having a loan in default as of Aug 2023, an indicator for whether the borrower was enrolled in an IDR (income-driven repayment) plan in Feb 2020 (prior to the repayment pause), an indicator for whether the borrower was signed up for auto debit in Feb 2020 (prior to the repayment pause), the % of months the borrower ever had a loan payment past due for 30 days or more (30-day delinquency) from January 2015 to March 2020 (prior to the repayment pause), and indicators for whether the participants first missed a payment on all possible missed payment dates (prior to eligibility for study participation being determined and prior to receiving an intervention treatment email). If a control variable contained missing data, we addressed it in two steps: i) for binary or categorical variables, missing values were replaced with zero; for continuous variables, missing values were replaced with the mean that variable took on in non-missing observations; ii) we added an indicator variable to the regression, coded as one if the original variable was missing and zero otherwise. For Model 1, borrowers who had already signed up for a Saving on a Valuable Education (SAVE) plan as of the send date of our first intervention email message and borrowers who were ineligible to apply for a SAVE plan were excluded from our analysis. For Model 2, borrowers who had signed up for auto debit on all their loans as of the send date of our first intervention email message were excluded from our analysis. All sample sizes reported above are rounded to the nearest hundred borrowers in accordance with disclosure policies required by the Department of Education.

Table S7. Regression-estimated impact of describing IDR benefits in % (vs. $) terms and recommending two recommended actions repeatedly (vs. one of two recommended action sequentially) on a borrower applying to an IDR plan within 30 days of receiving intervention (Model 1), signing up for auto debit within 30 days of receiving intervention (Model 2), making at least one payment within 30 days of receiving intervention (Model 3), and entering into 60-day delinquency within 30 days of receiving intervention (Model 4).

	**Model 1**	**Model 2**	**Model 3**	**Model 4**
IDR Benefits Described in %	0.00170***	-0.00004	0.00010	-0.00001
	(0.00012)	(0.00009)	(0.00023)	(0.00001)
Two-Action Email	0.00253***	-0.00014	-0.00080***	-0.00000
	(0.00012)	(0.00009)	(0.00023)	(0.00001)
Are Controls Included?	Yes	Yes	Yes	Yes
Number of Observations	8,442,700	9,796,300	10,008,800	10,008,800
Adjusted R-Squared	0.01	0.03	0.13	0.01

*** $p < 0.001$, ** $p < 0.01$, * $p < 0.05$, $^{+}$ $p < 0.10$

Notes: This table reports the results of four ordinary least squares (OLS) regressions including the subset of borrowers assigned to one of four conditions in our experiment: the "Two-Action, % IDR Benefits Email with Reminder" condition, the "Two-Action, $ IDR Benefits Email with Reminder" condition, the "One-Action, % IDR Benefits Email with Reminder" condition and the "One-Action, $ IDR Benefits Email with Reminder" condition. The dependent variables are a binary indicator for whether a borrower applied for an IDR plan within 30 days of first receiving an intervention email (Model 1), a binary indicator for whether a borrower signed up for auto debit within 30 days of first receiving an intervention email (Model 2), a binary indicator for whether a borrower made at least one loan payment within 30 days of first receiving an intervention email (Model 3), and a binary indicator for whether a borrower lapsed into 60-day delinquency within 30 days of first receiving an intervention email. The primary predictor variables are indicators for whether a borrower received communications that described IDR benefits in % (vs. $) terms and whether a borrower received communications that recommended two actions repeatedly (vs. communications that recommended one of two recommended actions sequentially). For all models, control variables were included for borrower age as of Aug 2023, an indicator for whether the borrower was male, indicators for education grade level reported on the borrower's last FAFSA application as of Aug 2023, an indicator for whether the borrower graduated from their last reported educational institution attended as of Aug 2023, indicators for the borrower's state of residence as of Aug 2023, the number of days that elapsed between when a borrower took out their last loan as of Aug 2023 and when they first received an intervention email, the total funds loaned to the borrower as of Aug 2023, the borrower's current outstanding loan balance as of Aug 2023, the borrower's monthly loan payment amount owed as of Aug 2023, an indicator for a borrower's loan servicer as of Aug 2023, an indicator for the borrower's last loan repayment type prior to the repayment pause as of March 2020 (e.g., standard repayment plan, graduated repayment plan, etc.), indicators for each loan type owned by borrower as of Aug 2023 (i.e., Parent PLUS loans, Consolidation loans), an indicator for ever having a loan payment past due for 60 days or more (60-day delinquency) as of Aug 2023, an indicator for ever having a loan in default as of Aug 2023, an indicator for whether the borrower was enrolled in an IDR (income-driven repayment) plan in Feb 2020 (prior to the repayment pause), an indicator for whether the borrower was signed up for auto debit in Feb 2020 (prior to the repayment pause), the % of months the borrower ever had a loan payment past due for 30 days or more (30-day delinquency) from January 2015 to March 2020 (prior to the repayment pause), and indicators for whether the participants first missed a payment on all possible missed payment dates (prior to eligibility for study participation being determined and prior to receiving an intervention treatment email). If a control variable contained missing data, we addressed it in two steps: i) for binary or categorical variables, missing values were replaced with zero; for continuous variables, missing values were replaced with the mean that variable took on in non-missing observations; ii) we added an indicator variable to the regression, coded as one if the original variable was missing and zero otherwise. For Model 1, borrowers who had already signed up for a Saving on a Valuable Education (SAVE) plan as of the send date of our first intervention email message and borrowers who were ineligible to apply for a SAVE plan were excluded from our analysis. For Model 2, borrowers who had signed up for auto debit on all their loans as of the send date of our first intervention email message were excluded from our analysis. All standard errors shown are HC1 robust standard errors. All sample sizes reported above are rounded to the nearest hundred borrowers in accordance with disclosure policies required by the Department of Education.

Table S8. Regression-estimated impact of describing IDR benefits in % (vs. $) terms and recommending two recommended actions repeatedly (vs. one of two recommended action sequentially) on a borrower applying to an IDR plan within 60 days of receiving intervention (Model 1), signing up for auto debit within 60 days of receiving intervention (Model 2), making at least one payment within 60 days of receiving intervention (Model 3), and entering into 60-day delinquency within 60 days of receiving intervention (Model 4).

	**Model 1**	**Model 2**	**Model 3**	**Model 4**
IDR Benefits Described in %	0.00248***	-0.00005	0.00059*	-0.00145***
	(0.00015)	(0.00010)	(0.00025)	(0.00027)
Two-Action Email	0.00294***	-0.00007	-0.00060*	-0.00100***
	(0.00015)	(0.00010)	(0.00025)	(0.00027)
Are Controls Included?	Yes	Yes	Yes	Yes
Number of Observations	8,442,700	9,796,300	10,008,800	10,008,800
Adjusted R-Squared	0.02	0.04	0.17	0.19

*** $p < 0.001$, ** $p < 0.01$, * $p < 0.05$, + $p < 0.10$

Notes: This table reports the results of four ordinary least squares (OLS) regressions including the subset of borrowers assigned to one of four conditions in our experiment: the "Two-Action, % IDR Benefits Email with Reminder" condition, the "Two-Action, $ IDR Benefits Email with Reminder" condition, the "One-Action, % IDR Benefits Email with Reminder" condition and the "One-Action, $ IDR Benefits Email with Reminder" condition. The dependent variables are a binary indicator for whether a borrower applied for an IDR plan within 60 days of first receiving an intervention email (Model 1), a binary indicator for whether a borrower signed up for auto debit within 60 days of first receiving an intervention email (Model 2), a binary indicator for whether a borrower made at least one loan payment within 60 days of first receiving an intervention email (Model 3), and a binary indicator for whether a borrower lapsed into 60-day delinquency within 60 days of first receiving an intervention email. The primary predictor variables are indicators for whether a borrower received communications that described IDR benefits in % (vs. $) terms and whether a borrower received communications that recommended two actions repeatedly (vs. communications that recommended one of two recommended actions sequentially). For all models, control variables were included for borrower age as of Aug 2023, an indicator for whether the borrower was male, indicators for education grade level reported on the borrower's last FAFSA application as of Aug 2023, an indicator for whether the borrower graduated from their last reported educational institution attended as of Aug 2023, indicators for the borrower's state of residence as of Aug 2023, the number of days that elapsed between when a borrower took out their last loan as of Aug 2023 and when they first received an intervention email, the total funds loaned to the borrower as of Aug 2023, the borrower's current outstanding loan balance as of Aug 2023, the borrower's monthly loan payment amount owed as of Aug 2023, an indicator for a borrower's loan servicer as of Aug 2023, an indicator for the borrower's last loan repayment type prior to the repayment pause as of March 2020 (e.g., standard repayment plan, graduated repayment plan, etc.), indicators for each loan type owned by borrower as of Aug 2023 (i.e., Parent PLUS loans, Consolidation loans), an indicator for ever having a loan payment past due for 60 days or more (60-day delinquency) as of Aug 2023, an indicator for ever having a loan in default as of Aug 2023, an indicator for whether the borrower was enrolled in an IDR (income-driven repayment) plan in Feb 2020 (prior to the repayment pause), an indicator for whether the borrower was signed up for auto debit in Feb 2020 (prior to the repayment pause), the % of months the borrower ever had a loan payment past due for 30 days or more (30-day delinquency) from January 2015 to March 2020 (prior to the repayment pause), and indicators for whether the participants first missed a payment on all possible missed payment dates (prior to eligibility for study participation being determined and prior to receiving an intervention treatment email). If a control variable contained missing data, we addressed it in two steps: i) for binary or categorical variables, missing values were replaced with zero; for continuous variables, missing values were replaced with the mean that variable took on in non-missing observations; ii) we added an indicator variable to the regression, coded as one if the original variable was missing and zero otherwise. For Model 1, borrowers who had already signed up for a Saving on a Valuable Education (SAVE) plan as of the send date of our first intervention email message and borrowers who were ineligible to apply for a SAVE plan were excluded from our analysis. For Model 2, borrowers who had signed up for auto debit on all their loans as of the send date of our first intervention email message were excluded from our analysis. All standard errors shown are HC1 robust standard errors. All sample sizes reported above are rounded to the nearest hundred borrowers in accordance with disclosure policies required by the Department of Education.
